# Nanocellulose-Graphene Hybrids: Advanced Functional Materials as Multifunctional Sensing Platform

**DOI:** 10.1007/s40820-021-00627-1

**Published:** 2021-03-17

**Authors:** Abdelrahman Brakat, Hongwei Zhu

**Affiliations:** State Key Lab of New Ceramics and Fine Processing, School of Materials Science and Engineering, Tsinghua University, Beijing, 100084 People’s Republic of China

**Keywords:** Nanocellulose, Graphene, Nanocomposites, Hybrid materials, Multi-sensing

## Abstract

Naturally derived nanocellulose with unique physiochemical properties and giant potentials as renewable smart nanomaterials opens up endless novel advanced functional materials for multi-sensing applications. However, integrating inorganic functional two-dimensional carbon materials such as graphene has realized hybrid organic–inorganic nanocomposite materials with precisely tailored properties and multi-sensing abilities. Altogether, the affinity, stability, dispersibility, modification, and functionalization are some of the key merits permitting their synergistic interfacial interactions, which exhibited highly advanced multifunctional hybrid nanocomposites with desirable properties. Moreover, the high performance of such hybrids could be achievable through green and straightforward approaches. In this context, the review covered the most advanced nanocellulose-graphene hybrids, focusing on their synthetization, functionalization, fabrication, and multi-sensing applications. These hybrid films exhibited great potentials as a multifunctional sensing platform for numerous mechanical, environmental, and human bio-signals detections, mimicking, and in-situ monitoring.

## Highlights

- The current status, ongoing challenges, and potential future outlooks of nanocellulose-graphene hybrids in multi-sensing applications are highlighted.
- The fundamentals of synthetization, interfacial interactions, functionalization, and green fabrication techniques of nanocellulose-graphene hybrids are described.
- The most advanced novel nanocellulose-graphene hybrids implementation as a multifunctional sensing platform is discussed.

## Graphical abstract

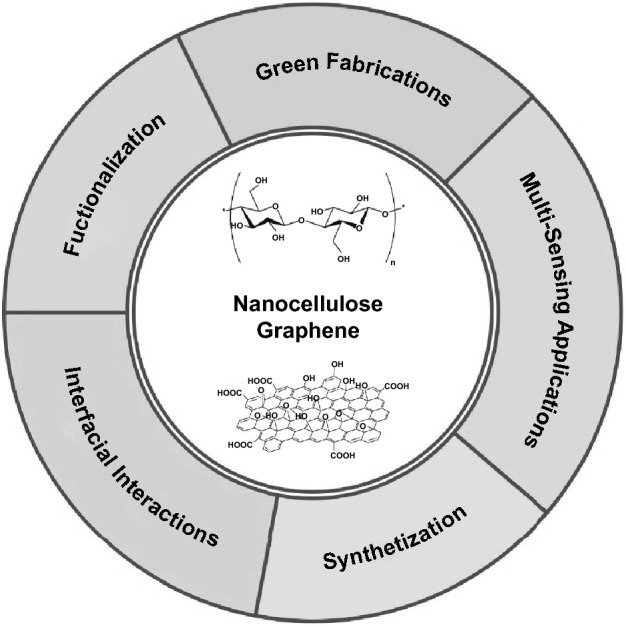

## Introduction

Recently, hybrid organic–inorganic nanocomposites synthesized and fabricated through eco-friendly approaches using renewable smart materials and inorganic functional materials have attracted significant attention due to numerous merits and unique properties. Since there has been rising concern about dependency on finite and exhausting non-renewable resources. In essence, designing and developing such hybrid materials have been a key principle for next-generation advanced functional materials and environmental sustainably. Renewable smart materials display intelligent behavior in response to different stimuli such as stress–strain, light, humidity, temperature, electrical input, pH, and magnetic force [1–4]. Nanocellulose (NCs) is one of the most attractive renewable smart materials owing to its unique properties such as stability, low coefficient of thermal expansion, and renewability [5, 6]. Moreover, the non-toxicity, biocompatibility, biodegradability, and eco-friendliness merits of nanocellulose in nature considered it as a potential renewable green substrate/fillers in nanocomposites with no side effects on health and environment, which will significantly wide broaden their applications [7]. Nanocellulose can be classified into three main forms, including cellulose nanocrystals (CNCs), cellulose nanofibrils (CNFs), and bacterial nanocellulose (BNCs) through mechanical–chemical treatments with different morphologies, sizes, and other properties. However, all these types have the same chemical composition. NCs have a great potential to act as stable carrier and matrix for multifunctional nanocomposites eco-friendly synthesis based on the derivative of forming a hybrid film with stiff and robust chain homomolecular structures [3, 8]. Mainly, it can serve as reinforcing filler in nanocomposites and a handle for adding stimuli responsiveness. The hydrophilicity–hydrophobicity originated from the enriched O–H and C–H, respectively, and hydrogen bonding between hydroxyl groups and oxygen-containing groups have made NCs an ideal candidate for the fabrication of two-dimensional carbon materials [8–10]. As a means in the direction of the realization of further improve the functionality of renewable smart materials, hybrid nanocomposites of inorganic functional materials are familiarized by incorporating functional two-dimensional carbon materials and green solvents [11]. Among various two-dimensional carbon materials, graphene exhibits the potential to be an ideal fabrication platform for innovative hybrid nanocomposites. Essentially, graphene oxide (GO) as unique derivatives of graphene through chemical exfoliation of graphite with a negative charge and oxygen-containing functional groups (i.e., hydroxyl, carboxyl, carbonyl, and epoxide groups) on basal planes, centers, and *sp*^2^ carbon sheets edges, ultimately enhanced the stability and dispersibility in the polar solvent [12–14]. It can provide the possibility and functionality of designing different smart hybrid materials as sensors based on their desirable properties such as extra-ordinary electrical-thermal conductivity, high surface area, binding potential, and aqueous processability [15–17]. However, GO nanosheets aggregation is caused by van der Waals forces resulting in inferior mechanical properties [18].

Interestingly, studies have been found that NCs can assist dispersion and stability and enhance the interlayer interaction between GO nanosheets [19]. The necessity of modification is attributable to the GO hard dispersibility in conventional solvents, limiting the adaption in practical application [20–24]. Also, ionic liquids (ILs) as green solvent are considered as a new and versatile platform for the dissolving, dispersion, and modifying of various nanomaterials, including NCs and GO due to their combined unique properties as high chemical–thermal stability, high ionic-electrical conductivity, non-flammability, non-volatility, and ease of recycling [24–26]. The multifunctional role of ILs can develop the hybrid nanocomposites represented in incorporating functional groups with large cation/anion combinations [27, 28]. Consequently, ILs can serve as dispersant, stabilizer and precursor for NCs-GO hybrid nanocomposites. Accordingly, it can offer an attractive eco-synthetic route of NCs-GO hybrid nanocomposite, which turns it a promising candidate for green fabrication of regenerated hybrid materials [20–22, 26, 27]. A list of functional nanocellulose-graphene hybrids with outstanding rheological, mechanical, electrical, dielectric, thermal, and optical properties was reported by combining modified nanocellulose chains with graphene sheets in a well-controlled manner as represented in Table 1 [29–39]. Simple solution-based methods and multilayer assembly techniques have been widely used to fabricate these hybrid nanocomposites because it is a facile and eco-friendly approach for diverse applications such as multi-sensing. The simplicity, versatility, and nanoscale structural control merits of these techniques considered it an ideal platform for the fabrication of NCs chains and graphene sheets into hybrid films with unique properties and multifunctionality [40, 41]. In this present review, we highlight the current status of nanocellulose-graphene hybrids in multi-sensing applications. As outlined, the basic principles of synthetization, interfacial interactions, and functionalization of these hybrid nanocomposites are described. Subsequently, we summarize the recent progress in the fabrication techniques of regenerated hybrid films. Afterward, we intensely focus on the various utilizing such hybrid films as a multifunctional sensing platform. Eventually, ongoing challenges and potential future outlooks regarding nanocellulose-graphene hybrids are highlighted and briefly discussed.

**Table 1:** Tabulation of the synthetization, functionalization, fabrication, and properties for a variety of hybrid NCs-GO/rGO nanocomposite films

Hybrid nanocomposites description	Synthetization–functionalization method	Fabrication technique	Hybrid film potentials	References
CNFs-GO	GO chemical reduction via hydrazine reagent	Simple filtration	Portable and bendable electronic devices	Luong et al. [30]
NCs-GO	[Bmim]Cl ionic liquid	Multilayer immersive assembly	Sensors and electrochemical devices	Tang et al. [105]
NCs-GO	DMAc/LiCl solvent	Simple blending drying	High-performance	Zhang et al. [38]
BNCs-GO	GO chemical reduction via hydrazine reductant	Simple vacuum-assisted self-assembly	Advanced electrochemical devices	Feng et al. [142]
CNCs-GO	GO thermal reduction	Drop-casting Vacuum drying	Exciting functional properties	Valentini et al. [133]
CNCs-GO	Isophorone diisocyanate (IPDI) coupling reagent GO reduction via hydrazine reductant	Multilayer spraying assembly	Proximity Photoelectric sensor Detecting finger approaches	Sadasivuni et al. [146]
NCs-GO	DMAc/LiCl solvent GO thermal reduction	Drop-casting Vacuum drying	Simple, flexible, and efficient temperature sensor T: (25–80 °C)	Sadasivuni et al. [31]
CNCs-GO	NCs functionality and synergistic interaction	Simple blending-casting drying	Renewable, flexible, and cheap humidity sensor RH: (25–90%)	Kafy et al. [34]
CNCs-GO	Polyanion/polycation surface recharge (−/ +) modification Polyethyleneimine (PEI) GO electrochemical reduction	Multilayer spin assembly	Wearable electronic devices	Xiong et al. [106]
CNFs-GO	Orientation-direction control and alignments GO thermal reduction	Multilayer deposition assembly	Anisotropic thermally conductive (CNFs-rGO)_40_ layers Applications in thermal management	Song et al. [96]
CNFs-GO	GO thermal reduction Structure alignment between hydrophobic rGO and acetate CNFs hydrophilic substrate	Electrospinning-deposition assembly Hot pressing	Human finger bending and pushing motions sensor Strain sensor	Fu et al. [130]
CNFs-GO	[Amim]Cl ionic liquid	Simple blending-casting-drying	Novel multifunctional hybrid film	Zhang et al. [136]
CNFs-GO	GO Green reduction with HI reductant	Step by step vacuum filtration	Biobased flexible electronic devices	Hou et al. [155]
NCs-GO	GO green chemical reduction via HI reductant	Drop casting drying	Multifunctional wearable sensor Simultaneous in situ monitoring of human motion and sweat	Xu et al. [165]
NCs-GO	Alkaline–aqueous urea solution In situ GO chemical reduction via L-ascorbic acid reductant	Blending casting drying	Multifunctional Sensor with high sensitivity to different stimuli’s: mechanical, environmental, and human bio-signals	Chen et al. [32]
CNFs-GO	GO chemical and thermal reduction via reagents (Vitamin C and HI) green reductants	Blending filtration drying	Portable and wearable electronic devices	Chen et al. [37]

## Materials Synthesis and Properties

In this section, we focus on the essential synthetization routes of the nanocellulose and graphene as their classification, preparation, dimension, and modifications, including their unique properties. In essence, these potential characteristics will clarify the interfacial interactions, specificity, and superiority of nanocellulose chains and graphene sheets as a multifunctional sensing platform.

### Nanocellulose

**Fig. 1:**
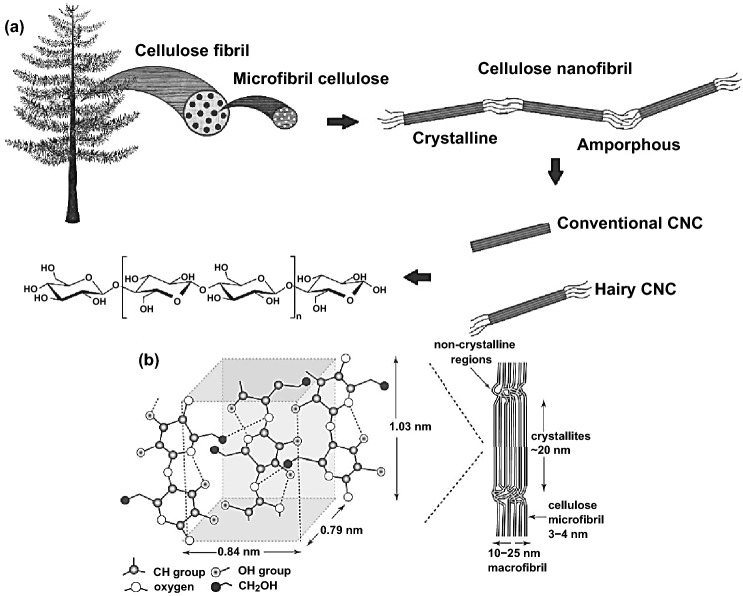
**a** Schematic representation of top-down cellulose hierarchical structure from origin source. Reproduced with permission from Ref. [166] Copyright © 2020, Springer Nature. **b** Intra-intermolecular strong hydrogen bonding cellulose structure and glucose monomers alternately rotated degree (180°) with crystalline-non-crystalline regions. Reproduced with permission from Ref. [167] Copyright © 2012, The Royal Society

Nanocellulose, as one of the most abundant linear biopolymers on the earth, is typically categorized into forms of cellulose nanocrystals (CNCs), cellulose microfibrillated (CNFs), and bacterial nanocellulose (BNCs), referred to the extraction routes from the hierarchical structure and origin sources as illustrated in Fig. 1a by various mechanical and chemical treatments [9]. The NCs characteristics and properties are dependent on the processing parameters and natural origin. Since varied origin sources (i.e., softwood, hardwood, plants, and others) are complex components of cellulose, hemicellulose, and lignin, further treatments and purifications are needed to obtain nanocellulose [9, 42]. The NCs derivated macromolecules offered unique mechanical, optical, thermal, and electrical properties attributes to the chemical stability, compatibility, and hydrophobic–hydrophilic features [5].

**Fig. 2:**
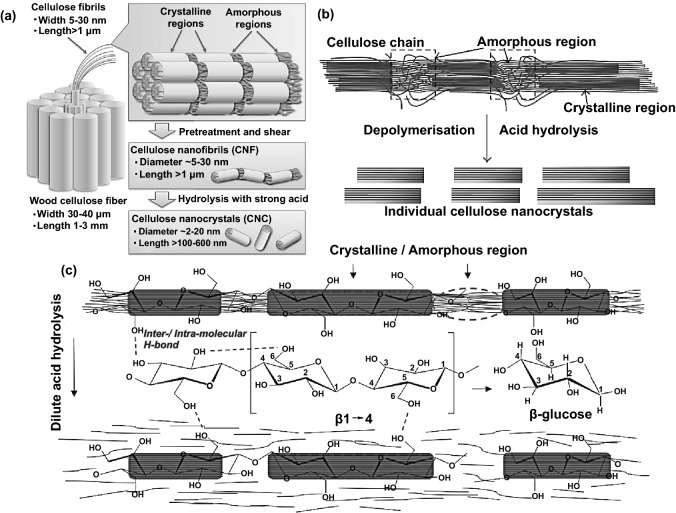
**a** Schematic view of nanocellulose obtained from wood via pretreatment and post-treatment of cellulose. Reproduced with permission from Ref. [49] Copyright © 2016, American Chemical Society. **b** The representation of depolymerization of cellulose to nanocellulose. Reproduced with permission from Ref. [168] Copyright © 2014, Hindawi Publishing Corporation. **c** Schematic view of top-down acid hydrolysis of cellulose to separate crystalline regions from non-crystalline regions under the effect of intra-intermolecular hydrogen bonding. Reproduced with permission from Ref. [46] Copyright © 2015, Elsevier

NCs are nothing more than nanoscale linear biopolymer chains containing glucose monomers (cellobiose repeating unit), with 1D/2D/3D multidimensional unique structure merits, which refer to the monomer alternately rotated degree (180°) with its neighboring. The comprehensive networks of intra-intermolecular hydrogen bonding, as illustrated in Fig. 1b, as results of the abundant surface functional hydroxyl groups, make it highly flexible and robust. The cellulose structure variation from nanowhiskers, nanoparticles, nanofibers, nanocrystals, and microcrystalline depend on the degree of crystallinity and polymerization be determined by its origin and treatments related to the highly ordered crystalline and disordered non-crystalline amorphous regions (Fig. 2a, b) [43, 44]. Although the NCs’ chemical structures are the same, the physical and chemical properties are noticeably dissimilar. The extraction of nanoscale pure cellulose from macro–microlignocellulose sources could be achievable by eliminating lignin, hemicellulose, and other impurities through several mechanical and chemical treatments [9, 45]. Precisely, cellulose nanocrystals and cellulose nanofibrils can be extracted by separating the crystalline–amorphous regions through the top-down acid hydrolysis approach as a widely used chemical treatment [46, 47]. The crystalline regions remain undamaged due to their high acid hydrolysis resistance, while the non-crystalline amorphous domains are dispersed because they are highly susceptible to the acid hydrolysis in the amorphous chain dislocations as illustrated in Fig. 2c. Afterward the hydrolyzation process, NCs suspension is needed extra post-treatments such as quenching, washing, acid elimination, filtration, stabilization, modification, homogenization, centrifugation, ultrasonication, dialysis, purification, neutralization, and lyophilization, to obtain the final pure cellulose in different micro–nanoscale dimension and structures [9, 45, 47–49].

In the beginning, everyone was focused on the nanocomposite reinforcement because of the NCs lightweight (∼1.30 g cm^−3^), high strength (200–400 MPa), high elastic modulus (70–220 GPa) [50], high Young’s modulus (7.4–14 GPa) [51], and other mechanical properties. These outstanding mechanical properties considered it an efficient green reinforcing nanofiller compared with other artificial-synthetic fibers, wires, nanoparticles, nanotubes, and nanowhiskers [9, 52]. The NCs reinforcement efficiency relies on the isotropic–anisotropic morphological characteristics and high aspect ratio by transferring stresses and supporting nanocomposite matrix bonding [47, 53–56]. However, then it has shown great potentials in the field of smart materials and multiple sensing applications [5, 20, 57, 58]. Fundamentally, NCs’ highly reactive surface functional groups (i.e., hydroxyl) enable their conjunction via hydrogen bonding, ionic interactions, and van der Waals forces with numerous kinds of molecules, nanoparticles, and moieties for multi-sensing applications. Such abundant surface functional groups could be extending the capabilities of NCs-based hybrid nanocomposites with graphene sheets by providing active sites for wide-ranging chemical modifications of NCs. The nanosized high aspect ratio beyond 30 promotes dispersion and the synergistic surficial–interfacial interactions between NCs and other graphene derivatives (i.e., GO nanosheets). The large surface areas in a range of several hundred m^2^ g^−1^ [59, 60] as well considered it a unique multifunctional nanomaterial. More profoundly, NCs are not easily susceptible to thermal chemical degradation and decomposition at high temperature in the range of (200–400 °C) determining by the degree of crystallinity, presence of impurities and other physical–chemical factors [4, 9]. Interestingly, cellulose differsivity in macro-micro-nanoscale size, isotropic–anisotropic structure, and birefringence nature with varied refractive indices leads to remarkable optical phenomena [9, 61–63]. Likewise, NCs liquid crystallinity phase transformation and self-assembly into a layered structure with helicoidal chiral nematic ordered offering templating synthesis routes with other two-dimensional carbon materials precursor for next-generation advanced functional hybrid materials [64]. Such assembled macroscopic advanced materials with imparts tunable optical–photonic properties together with NCs swelling–shrinkage and expansion–contraction behaviors are considered as promising candidates in stimuli’s responsive and sensing applications as mechanochromic, thermochromic, and colorimetric [65, 66].

### Graphene and Graphene Oxide

**Fig. 3:**
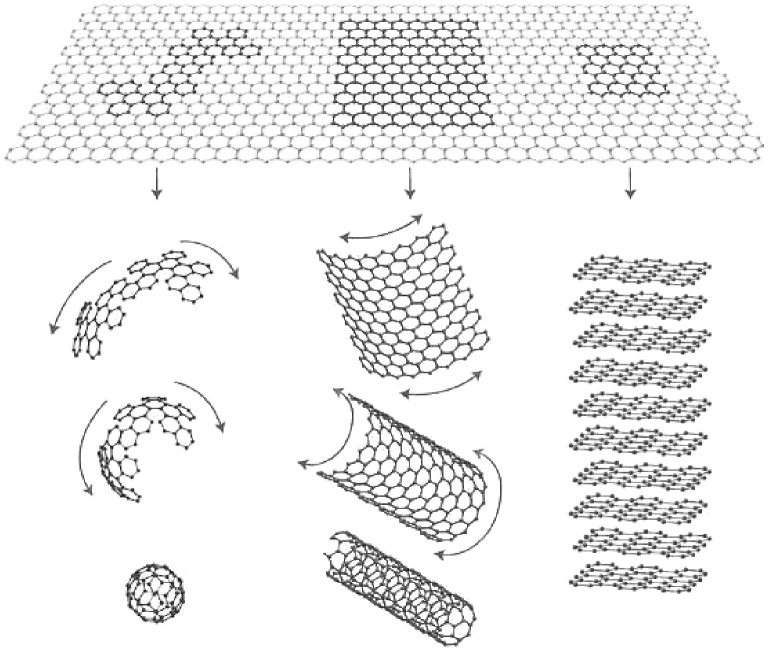
Schematic representation of graphene as the mother of all graphitic family and fundamental basis of all carbon materials dimensionalities. Reproduced with permission from Ref. [69] Copyright © 2007, Springer Nature

**Fig. 4:**
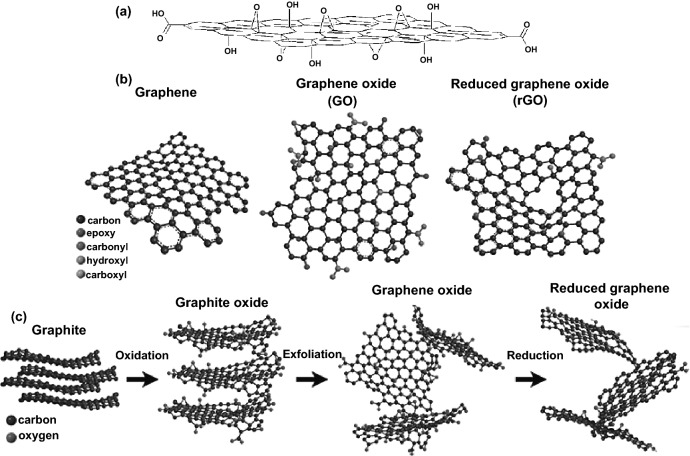
**a** Schematic model of GO basal–center–edge planes oxygen–containing functional groups. Reproduced with permission from Ref. [75] Copyright © 2008, American Chemical Society. **b** Schematic view of the graphene, graphene oxide, and reduced graphene oxide chemical structures. **c** Synthesized routes of graphite to graphene oxide and reduced graphene oxide. Reproduced with permission from Ref. [14] Copyright © 2016, Intech Open

Graphene is one of the two-dimensional carbon materials derivatives sheet-like materials discovered by Geim and Novoselov since 2004 with a flat monolayer of carbon atoms tightly packed into a two-dimensional crystalline hexagonal lattice and honeycomb structure due to the *sp*^2^ hybridization of carbon [67, 68]. It can be considered as the mother of the graphitic family and basic building block for all other dimensionalities of carbon allotropes materials (Fig. 3) [69]. It can also be categorized based on sheet layers, surface modifications, and oxygen content and orientation [70]. Since outstanding material offers a unique combination of superior properties as mechanical, electrical, thermal, optical, and electronic characteristics, thanks to their strong in-plane σ bonds and weak out-plane π bonds [71]. However, these outstanding features are only observed in graphene single defect-free layers, limiting their adaption in practical implementation [14]. Alternatively, among the various top-down synthesizing routes [72], the chemical exfoliation process of graphite oxide flakes into graphene oxide multilayer sheets using modified Hummers’ method [73] can be considered as a commonly used method with relative ease processing and cost-effective [74, 75]. GO is a highly oxidized synthesized version of graphene with unique single-multilayers sheets containing rich oxygen-containing functional groups (i.e., hydroxyl, carboxyl, carbonyl, and epoxy). Precisely, GO nanosheets mainly possess oxygen in the form of hydroxyl and epoxy functional groups at basal–center–edge planes with a minor contribution of decorative carboxyl and carbonyl functional groups at the plane of the edge as shown in Fig. 4a. These GO functional groups can be modifying the van der Waals interactions and affect its mechanical, electrical, and electrochemical features. More importantly, the GO amphiphilic is due to the hydrophilic oxygen-containing groups and hydrophobic aromatic frameworks, allowing its interactions with organic–inorganic molecules and functionalization [14, 76]. Furthermore, the following chemical, thermal, photocatalytic, and electrochemical reductions [77] of the GO oxygen content resulted in a partial restoration to graphene state and reduced graphene oxide (Fig. 4b).

It is worth noting that graphene’s oxidation and reduction process may lead to some impurities and structural defects that significantly influence the properties and characteristics. Even though GO and rGO have inferior properties compared with graphene, it can be modified and improved through covalent and non-covalent functionalization with moieties, molecules, and organics. Since active oxygen sites are much more significant in GO than rGO, GO is more reactive and suitable to be functionalized via covalent interactions. Conversely, rGO possesses a partial graphitic surface and initial structure with some oxygen active sites; consequently, making it adequate to be functionalized via non-covalent interactions [14]. Nevertheless, GO sheets’ highly hydrophobic and agglomeration tendency due to *π–π* interactions and van der Waals interactions between two-dimensional sheet layer lead to inferior their outstanding features. Therefore, GO-rGO can achieve tailored-designed properties with other green dispersants, stabilizers, and reductants such as nanocellulose, which expand the potential applications range. As previously mentioned, GO/rGO simple top-down synthesis has offered new possibilities for stimuli-response characteristics in nanocomposites. In addition, GO-rGO nanosheets exceptional mechanical, optical, electrical, and thermal properties have attracted significant attention in numerous applications such as sensing [78–81]. Two-dimensional carbon materials derivatives of monolayer graphene with well-reported significant mechanical properties include highly Young’s modulus (1.0 TPa) break strength (42 N m^−1^), and intrinsic tensile strength (130.5 GPa) [82]. GO/rGO monolayer could achieve these recorded properties with variation depending on the number of surface functional groups and defects from the synthetization process [83, 84]. Moreover, GO/rGO with highly basal-center-edge functional groups (i.e., –OH, –COOH) can be utilized as matrix/filler to enhance the mechanical properties and interfacial interactions of hybrid nanocomposites [85]. Turning to the most remarkable feature of graphene with two-dimensional layers of *sp*^2^ carbon one atom thick, which exceptionally possesses high electrical conductivity (6500 S m^−1^) [86] and high electron mobility (25 m^2^ V^−1^ s^−1^) [87].

Herein, it is worth mentioning that GO synthetization and fabrication process could make it electrically resistive (1.64 × 10^4^ Ωm) by disrupting the *sp*^2^ graphene orbitals bonding and movement charge carriers, which obstruct its electrical conductivity [88, 89]. Consequently, the necessity of GO reduction [39, 90, 91] is crucial to achieving desirable electrical conductivity [91, 92], which makes rGO a promising conductive filler candidates for hybrid nanocomposites. Similarly, monolayer graphene has shown exceptionally super-high in-plane thermal conductivity as high as ~ 2500–5000 W m^−1^ K^−1^ [71, 93]; however, synthesized GO has low thermal conductivity in the range of 0.5–1.9 W m^−1^ K^−1^ [94]. It has been reported that the GO reduction plays a critical role in terms of (in-plane *K* “*λ*_*X*_” and cross-plane *K*_⊥_ “*λ*_*Z*_”) thermal conductivity enhancement from around ~ 3 up to 61 W m^−1^ K^−1^ with *K*/*K*_⊥_ ratio ≈ 675 [94]. Profoundly, considering the non-negligible GO/rGO isotropic–anisotropic thermal conductivity behavior besides the controlling multilayers assembly in the direction of heat flow. In view of that, GO/rGO as matrix/filler has excellent potentials to achieve high thermal conductivities of hybrid nanocomposites films [95–97]. Also, GO possesses a number of notable optical properties as a consequence of its unique electronic configuration [98]. In contrast with pristine graphene (highly optical transparency of ∼ 97.7% in the visible range) [99], GO shows photoluminescence in various spectrum ranges from ultraviolet, visible, and near-infrared rely on its structure-dependent absorption [99]. Nevertheless, the transition from conducting to insulating state due to breaking graphene *sp*^2^ bond networks by GO oxygen functional groups can change the optical properties. Apparently, the combination of *sp*^2^ and *sp*^3^ hybridizations in GO breaks the graphene symmetry and opens up the gap band, making it promising candidates with optoelectronic properties [98, 100]. The GO thermal–chemical reduction process [101] and certain chemicals can partially restore graphene electronic properties and control the optical properties. Accordingly, desirable GO/rGO optical properties could be achievable through synthetization optimizing, reduction-deoxygenation, and multilayer assembly controlling of hybrid nanocomposites films.

## Synergistic Interfacial Interactions

Synergistic interfacial interactions are among the most crucial aspects of hybrid nanocomposites ideal design to achieve functional hybrid materials’ high performance. Based on the affinity, stability, and dispersibility between NCs chains and GO/rGO nanosheets ascribe to the hydrogen bonding and hydrophilic–hydrophobic interactions. Here we discuss the interfacial bonding-interactions which are relevant for the eco-synthesis of NCs-GO/rGO hybrid nanocomposites.

**Fig. 5:**
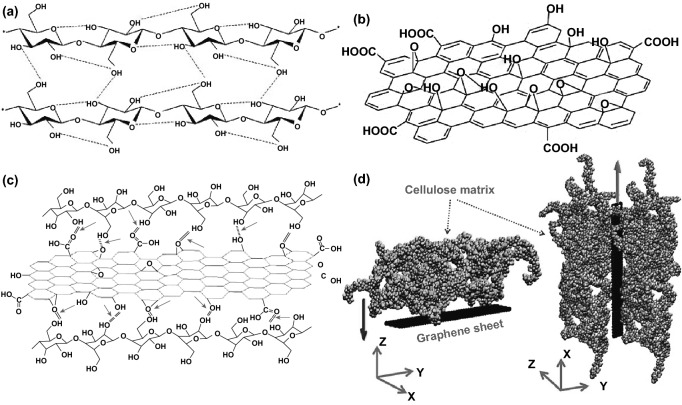
**a** NCs intra-intermolecular hydrogen bonding networks. Reproduced with permission from Ref. [102] Copyright 2018, Elsevier. **b** Graphene oxide proposed a structural model. Reproduced with permission from Ref. [104] Copyright © 2015, The Royal Society of Chemistry. **c** Schematic illustration of the hydrogen bonding (red rows) of regenerated GO nanosheets and NCs molecular chains. Reproduced with permission from Ref. [105] Copyright © 2012, Chinese Materials Research Society, Production and hosting by Elsevier B.V. **d** Model of the interfacial interactions between the graphene sheet and cellulose chain matrix. Reproduced with permission from Ref. [107] Copyright © 2013, American Chemical Society

As aforementioned, NCs possess abundant hydroxyl oxygen groups and strong intra-inter hydrogen bonding networks, making it stable and stiff [102], as demonstrated in Fig. 5a. Correspondingly, GO can be seen as two-dimensional amphiphilic molecular with hydrophobic π domains on its basal–center plane and hydrophilic –COOH groups on the edges, as shown in Fig. 5b [103]. This arrangement of hydrophobic–hydrophilic segments on the basal–center–edge planes allows the GO nanosheets to self-assemble and interact with NCs chains into a stable network structure with different covalent and non-covalent bonding as van der Waals interactions. Even though GO structure still an interesting researches object, but GO lattice can be interrupted by epoxide, hydroxyl, carbonyl, and carboxylic groups [104].

In 2012 as illustrated in Fig. 5c, Tang et al. [105] assumed that the hydrogen bonding interactions between the regenerated GO nanosheets and NCs chains could drive the composite deposition through layer-by-layer fabrication [96, 105, 106]. This assumption is based on the proven hydrogen bonding between oxidation functional groups of GO and the hydroxyl groups of NCs. Rahman et al. [107] have developed molecular modelling to determine the effect of weight concentrations, dispersion, and aspect ratio in cellulose-graphene hybrid nanocomposites interfacial properties. Figure 5d shows the interface interaction model of a single layer of graphene sheet with a cellulose chain matrix. It has been found that the cellulose-graphene interfacial interaction entirely dependent on the nonbonded interactions such as van der Waals terms and cellulose’s Young modulus enhanced the dispersion and in incorporating nanocellulose-graphene hybrid nanocomposites [107].

**Fig. 6:**
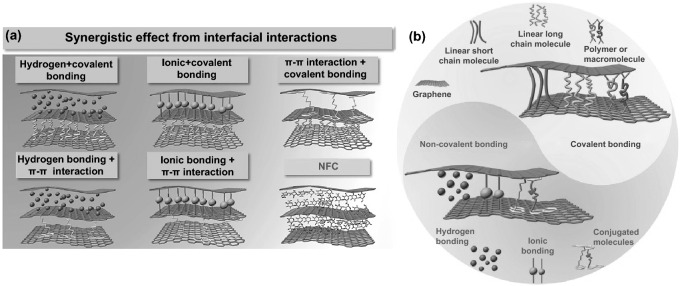
**a**, **b** Interfacial interaction categories in graphene-based nanocomposites, including non–covalent and covalent bonding. Reproduced with permission from Ref. [108] Copyright © 2017 Elsevier B.V

Interfacial interaction inspired by natural renewable smart materials through their micro–nanoscale hierarchical structure can gain functionality and achieve high-performance hybrid nanocomposites [96, 106, 108–111]. Gong et al. [108] discussed the potential enhancement in graphene-based nanocomposites’ mechanical properties inspired by the abundant interfacial interaction in natural materials through the covalent and non-covalent bonding between the graphene nanosheets as shown in Fig. 6a, b. It should be noted that the abundant surface functional groups present potentials for the adequate bonding of NCs chains to GO nanosheets via non-covalent as ionic bonding and hydrogen bonding. Consequently, the NCs intra-intermolecular chain can enhance crosslinking density with GO nanosheets through hydrogen bonding resulting in superior mechanical properties of hybrid nanocomposites. Synergistic interfacial interactions between NCs chains and GO nanosheets combine both covalent and non-covalent bonding, including π–π interactions and electrostatic interactions by modification of the surface functional groups charge (−/ +). For instance, Xiong et al. [106] found a synergistic ionic and hydrogen bonding effect between modified cationic cellulose nanocrystals networks and anionic GO nanosheets through multilayer assembly techniques, which leads to enhance mechanical properties.

## Functionalization

Functionalization and hybridization of nanocellulose-graphene hybrid nanocomposites are essential, including the assistant of NCs and ILs in GO/rGO nanosheets dispersion, stabilization, reduction, reinforcement, and functionalization.

### Nanocellulose as Multifunctional Green Dispersant, Stabilizer, Filler, and Reductant

Recently, nanocellulose has aroused a great deal of research interest as a multifunctional green dispersant, stabilizer, reinforcing filler, reductant, and functional template materials for numerous graphene derivatives (i.e., GO/rGO) due to their outstanding features. The dispersion of graphene derivatives in an eco-friendly manner is very attractive since green dispersants can be cost-effective in large-scale production and eco-effective in harmful chemical elimination. As a renewable material, nanocellulose extracted from abundant natural wood with 1D/2D/3D morphological, structural, surface modifications, and surface functionalization merits, making it an ideal green dispersant, stabilizer, and reductant for two-dimensional carbon materials derivatives. Solubility and processability are the first well-accepted dispersion options for various applications of graphene derivatives [32]. Graphene-based nanomaterials dispersion can be improved by reducing its precursor, which can gain functionality with numerous functional groups like hydroxyls, carbonyls, and epoxides. Consequently, this offers access to modify and enhance GO dispersion in the polar environment [112, 113].

**Fig. 7:**
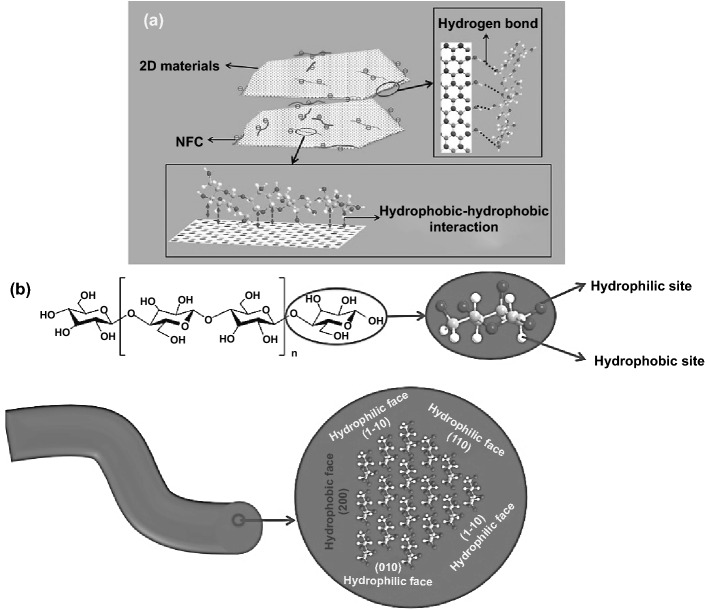
**a** Schematic view of two-dimensional materials disperse by Nanocellulose. **b** Schematic view of hydrophilic–hydrophobic crystalline faces of cellulose. **c** Three-dimension chemical structure of cellulose displays the hydrophilic–hydrophobic sites. Reproduced with permission from Refs. [19, 169]. Copyright © 2015, Elsevier

Nanocellulose plays a critical role to disperse GO owing to the presence of abundant functional groups such as polar hydroxyl group –OH [19, 114]. These numerous functional groups enable water binding and moisture sorption–retention possessions; thus, NCs display high water content and a wide range of applications [115]. Moreover, surface functional groups charge increasing the dispersion limit, which leads to extra electrostatic stabilization and prevents aggregation between the two-dimensional carbon materials and NCs chains [116]. The NCs dispersion mechanism of two-dimensional materials clarified by the interfacial interactions between the two-dimensional nanosheets and NCs chains, including the strong hydrogen bonding among NCs hydroxyl groups and two-dimensional materials into the edges [19]. As illustrated in Fig. 7, Li et al. [19] have been confirmed that the existence of hydrophobic interaction and hydrogen bonding between two-dimensional materials nanosheets and hydrophobic crystalline faces of NCs, is responsible for the excellent dispersibility and stability in hybrid nanocomposites with superior mechanical–electrical properties. Figure 7a shows the C, O, and H atoms with grey, red, and white spheres, separately, together with the negative surface charge (−) generated by NCs carboxyl group [19]. Correspondingly, Yu et al. [35] have used the NCs to help stabilize the dispersion of chemically reduced graphene oxide in the aqueous system depending on the hydrophobic interactions between GO nanosheets and NCs crystalline faces. In addition to acting as an effective dispersant and stabilizer, NCs attract more attention as renewable bioderived reductants in hybrid nanocomposites due to abundant surface functional groups that could grant it with reduction power [117, 118]. Particularly, NCs can deoxygenate exfoliated GO and bind or network with rGO nanosheets with strong synergistic interfacial interaction [105, 119, 120]. For example, Peng et al. [120] have reported an eco-friendly approach for simultaneous reduction and functionalization of GO using NCs as green reductant. Hence, NCs can be considered a suitable and eco-friendly dispersant, stabilizer, and reductant for GO without disposal solvents.

### Ionic Liquids as Multifunctional Green Solvent

The functionalization of nanocellulose-graphene hybrid nanocomposite directly results from the affinity and interconnectedness of its constituents; therefore, controlling surface functional groups is significant when developing the hybrid materials. The functionalization of NCs-GO is mostly dependent on the process of modification in the efficient solvent. As mentioned before, NCs dissolution and regeneration are vital aspects in nanocomposites hybridization. Recent studies have found that several ionic liquids (ILs) can dissolve the NCs in a much simpler and effective eco-friendly method [22, 121]. Swatloski et al. [26] have reported that ionic liquids (ILs) can be used as green solvents for cellulose. The dissolution mechanism of cellulose in ILs is based on the anions acceptors and cations donors.

**Fig. 8:**
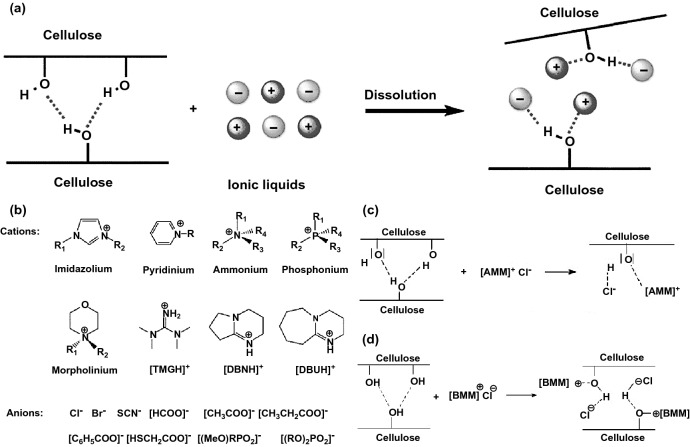
**a** Typical cations/anions based–ILs structures for dissolving NCs. **b** NCs dissolution mechanism in ILs. Reproduced with permission from Ref. [22] Copyright © 2017, American Chemical Society. **c** NCs dissolution mechanism in AmimCl. Reproduced with permission from Ref. [170] Copyright © 2005, American Chemical Society. **d** NCs dissolution mechanism in BmimCl. Reproduced with permission from Ref. [23] Copyright © 2009, American Chemical Society

**Fig. 9:**
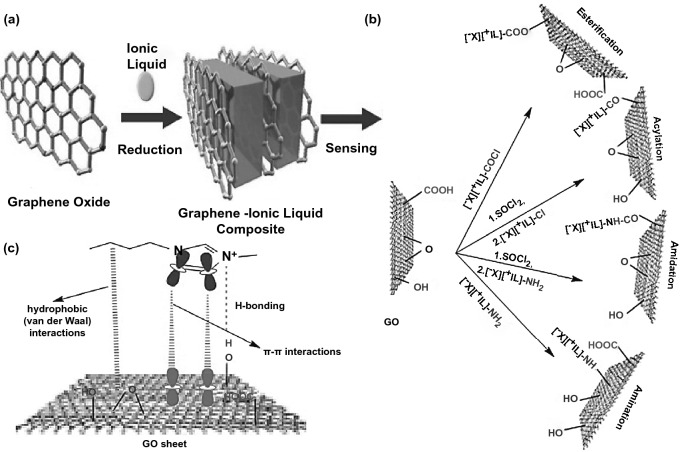
**a** Schematic view of the GO/rGO synthetization and functionalization via ILs for sensing applications. Reproduced with permission from Ref. [129] Copyright © 2010, Wiley‐VCH Verlag GmbH & Co. KGaA, Weinheim. **b** Schematic view of GO covalent functionalization. **c** Schematic view of GO non–covalent functionalization. Reproduced with permission from Ref. [21] Copyright © 2018, Springer Nature

Figure 8 demonstrates the NCs dissolution mechanism in ILs due to the disruption of the NCs hydrogen bonding networks through the electrostatic interactions caused by the ILs charges pieces. The electron donor–acceptor generated by NCs hydrogen (H) and oxygen (O) atoms congregation with the ILs anions and cations. ILs anions and cations act as hydrogen bond acceptor and hydrogen bond donor, respectively. It was reported that among these typical cations/anions based–ILs (Fig. 8b), 1-allyl-3-methyl-imidazolium chloride (AmimCl) and 1-butyl-3-methyl-imidazolium chloride (BmimCl) are the most suitable and favorite green solvent as shown in [25, 26, 105, 112, 122]. Zhang et al. [122] proved the hydrogen bonding interactions between the NCs hydroxyls groups and ILs anions-cations. However, it should be noted that NCs dissolution and regeneration by the ILs rely on several factors, including temperature, time, NCs origin, ILs structure, and ILs removal process [20, 23, 24]. On the other hand, the synthetization of GO thru ILs is gaining great attention due to the strong interfacial interactions and tunable physicochemical properties. ILs could be easily eco-friendly dispersed, stabilized, and reduced GO. GO-ILs stabilization, dispersion, and reduction mostly involve covalent and non–covalent donor–acceptor interactions and electrostatic forces [21, 123–125]. For instance, Zhang et al. [126] successfully prepared a stable dispersed and reduced GO by ILs without surface modification. It was suggested that *π–π* interaction, cation–π interaction and anion–*π* interaction between rGO nanosheets is responsible for the stabilization of rGO-ILs. GO can be covalent functionalized owing to the surface and edge interactions of abundant hydroxyl (–OH) and carboxyl (–COOH) functional groups (Fig. 9). Correspondingly, GO non-covalent functionalized attributed to the π–π interaction, repulsive and van der Waals forces between GO nanosheets without any influence in the electrical and thermal conductivity [21]. For example, Wang et al. [127] used ILs to reduce and functionalize GO through an environment-friendly facile approach, resulting in stabled and well-dispersed rGO-ILs composite with excellent electrochemical activity. Similarly, Xu et al. [128] prepared a highly sensitive electrochemical sensor based on IL functionalized GO nanocomposite to detect ascorbic acid. Covalent and non-covalent functionalization of GO, as illustrated in Fig. 9b, c, also can be eco-friendly achieved through ILs for using in multipurpose applications as multi-sensing and environmental remediation [129].

## Fabrication

Nowadays, smart hybrid materials fabrication by renewable resources and green preparation techniques has attracted critical attention amongst recent researchers. Research interest in utilizing nanocellulose in particularly developing such advanced functional hybrid materials is rising rapidly, partly due to their stabilizing characteristics, surface modifications, functionalization, and outstanding mechanical properties, besides the fabrication and processing of several other desirable features of hybrid films. As previously mentioned, NCs, as smart renewable materials with chemical modifications potential to desired physicochemical properties, can exhibit functionalization routes with surface modifiers, chemical linkers, and coupling agents in hybrid nanocomposites. The hydrophilicity and hydrophobicity originated from the enriched O–H and C–H functional groups, respectively, which have made NCs an ideal candidate for the fabrication of two-dimensional carbon materials. These NCs’ abundant functional groups allow it to interact with other functional groups to achieve tailored properties and regenerate hybrid films for multi-sensing applications. Correspondingly, GO can be considered a fabrication platform of innovative hybrid materials with numerous functional groups and extraordinary properties. It has been reported that there is a strong interfacial interaction between GO/rGO conductive layers and NCs hydrophilic substrates [96, 130]. However, GO reduction required reductants reagents (i.e., titanium trichloride, sodium borohydride, and hydrazine) [131, 132], which mostly are toxic and harmful for health and the environment. Therefore, extra surfactants and stabilizers are commonly required to avoid the irreversible aggregation of GO layers during the reduction process. Alternatively, green chemical reductant reagents such as L-ascorbic acid (Vitamin C) and hydroiodic acid (HI) with thermal reduction are considered an eco-friendly and feasible GO reduction method. Until now, researchers have been studying green and straightforward approaches to fabricate nanocellulose-graphene nanocomposites with stable morphology and good dispersion. In this regard, green organic polar solvents have been reported to enhance the dispersion of nanocellulose-graphene nanocomposites. In particular, ionic liquids (ILs) have been used as an effective green solvent to fabricate the regenerated nanocellulose-graphene hybrid nanocomposite [105, 120]. This section focuses on the most advanced green fabrication techniques of macro–micro–nanoscale NCs-GO/rGO hybrid nanocomposites films with characteristics of lightweight, ultrathin free-standing, and high durability, which is essential to develop flexible, wearable, portable, highly conductive, and sensitive sensors for multiple sensing applications.

### Simple Solution-Based Methods

**Fig. 10:**
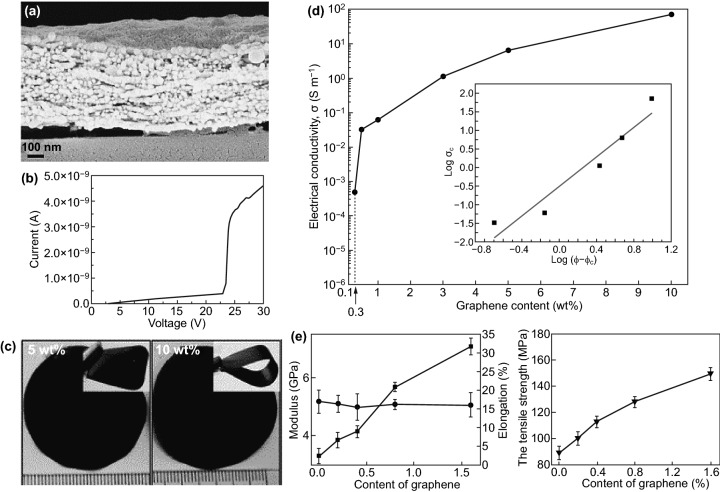
**a** CNCs-GO hybrid films morphological image. **b** The current–voltage curve of CNCs-GO hybrid films. Reproduced with permission from Ref. [133] Copyright © 2013, Elsevier Ltd. **c** Robust-conductive CNFs-rGO hybrid film. **d** rGO content influences electrical conductivity. Reproduced with permission from Ref. [30]. Copyright © 2011, The Royal Society of Chemistry. **e** GO content influences cellulose-GO Young’s modulus and tensile strength. Reproduced with permission from Ref. [38] Copyright © 2011 Elsevier Ltd

Several functional smart hybrid materials based on nanomaterial-incorporated nanocellulose-graphene have been fabricated through simple solution processes, including mixing, curing, and casting following by vacuum filtration drying. Valentini et al. [133] have fabricated homogeneous and conductive CNCs-GO hybrid nanocomposite film through simple mixing-casting methods. It found that the dispersibility and homogeneity due to the hydrogen bonding between GO abundant oxygen-containing groups with hydroxyl groups and oxygen atoms in CNCs chain (Fig. 10a). Moreover, the deoxygenation and thermal reduction of GO are responsible for the conductivity of the hybrid film (Fig. 10b). Similarly, Kafy et al. [34] have developed homogenous CNCs-GO hybrid nanocomposites and fabricated flexible humidity sensor using a simple blending-drying method. The reinforcement and well dispersion of GO in the CNCs matrix were proved based on the smoothness and non-agglomeration with high GO content (10 wt%). Luong et al. [30] have chemically reduced GO and combined with functionalized CNFs suspension in hydrazine (ammonia) solution [2, 30, 91]. In this work, they found that GO/rGO functional groups interacted with amine via covalent bonding, creating strong bonding and cross-linking bonds between rGO nanosheets and CNFs chains. Through a simple filtration method, robust CNFs-rGO hybrid film was fabricated with high tensile strength (273 MPa), as shown in Fig. 10c. Significantly, a high electrical conducting value (71.8 S m^−1^) was achieved with 10% of graphene content, as noted in Fig. 10d [30]. Likewise, Zhang et al. [38] prepared regenerated cellulose-GO nanocomposite film by incorporating dispersed GO into dissolved and modified cellulose matrix with the assistant of DMAc/LiCl solution [5, 38, 134, 135]. The stable cellulose-GO suspension and homogeneous blended nanocomposite film imputed to the well dispersion and strong interaction of GO nanosheets in the cellulose matrix. Also, it can be noted that the GO content has a significant influence on the apparent improvement of the mechanical properties of regenerated GO/cellulose hybrid composite film as appeared in the boost Young’s modulus (7.2 GPa) and tensile strength (148 MPa) in Fig. 10e [38]. As well, Sadasivuni et al. [31] reported a flexible and transparent NCs-rGO hybrid nanocomposite film as temperature and liquids sensor fabricated with the assistant of DMAc/LiCl solution and thermal annealing reduction of GO. Notably, rGO content significantly impacts the hybrid film’s electrical conductivity due to the carbon *sp*^2^ hybridization network and particle connection via GO chemical reduction. However, the uncompleted reduction of GO has an impact on the electrical conductivity performance of regenerated cellulose-GO hybrid film compared with what was achieved in the cellulose-rGO hybrid film by Luong et al. [30].

**Fig. 11:**
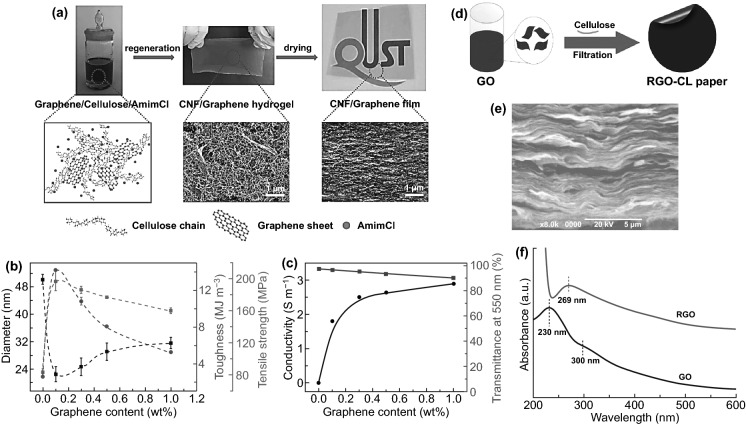
**a** Schematic view of in-situ regeneration of CNFs-Graphene hybrid nanocomposite via [Amim]Cl. **b** Mechanical properties of CNFs-Graphene hybrid film with CNFs dimension and GOs content. **c** Electrical conductivity and optical transparency of CNFs-Graphene hybrid film with different GO content. Reproduced with permission from Ref. [136] Copyright © 2017 American Chemical Society. **d** Schematic view of eco-friendly reduction of GO via [Bmim]Cl and combined with NCs. **e** SEM microstructural images of a fabricated NCs-rGO hybrid film by vacuum filtration with [Bmim]Cl. **f** UV−Vis absorption spectra of GO (0.1 mg mL^−1^) before and after the reduction with NCs. Reproduced with permission from Ref. [120] Copyright © 2012, American Chemical Society

ILs have been used to assist the fabrication of NCs-GO/rGO hybrids films due to the multifunctional role in synthesizing, reducing, and functionalizing such hybrids nanocomposites. As such, ILs multifunctional is more effective for green fabrication NCs-GO/rGO nanocomposites. Zhang et al. [136] demonstrated the first in-situ regeneration of CNFs-Graphene hybrid nanocomposite via 1-Allyl-3-methylimidazolium chloride [Amim]Cl, as illustrated in Fig. 11a. The resulting CNFs-Graphene hybrid films were flexible, transparent, and conductive with the potential of large-scale applications. These multifunctional hybrid films showed tailorable mechanical–electrical performance and transparency abilities attributed to the induced hydrophobic interactions between graphene nanosheets and cellulose chains, which generated a dense 3D network structure. Notably, graphene content significantly influences the mechanical properties and electrical conductivity (2.8 S m^−1^) besides the optical transparency (90.4%) of the re-generated hybrid film with 1.0 wt% graphene compared with pure CNFs (97.7%) Fig. 11b, c. Also, Peng et al. [120] have used 1-Butyl-3-methylimidazolium chloride [Bmim]Cl to reduced GO and fabricate flexible and conductive NCs-rGO hybrid film by vacuum filtration technique (Fig. 11d). Evidently, the highly conjugated electronic NCs-rGO hybrid lamellar structure (Fig. 11e) can be considered as direct evidence of the NCs and ILs reduction and functionalization of GO. NCs and ILs deoxygenation reduction of GO nanosheets were confirmed by the red shifting of the peak at 231 to 269 nm after reduction (Fig. 11f), besides removing the oxygen-containing functional group.

**Fig. 12:**
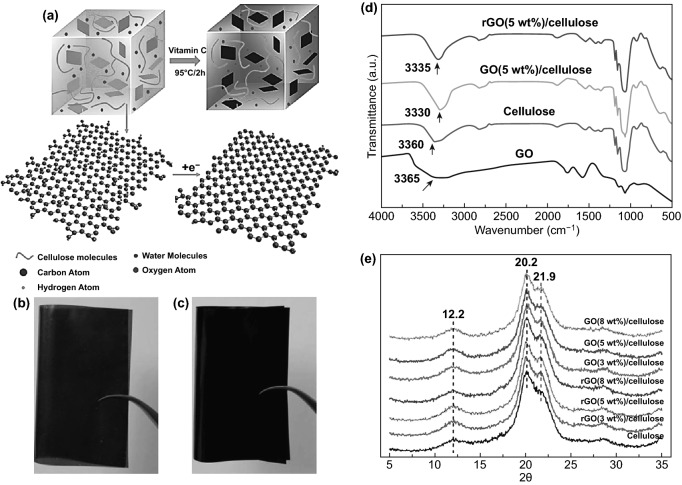
**a** Schematic view of in-situ GO chemical reduction in NCs matrix with L-ascorbic acid solution (Vitamin C). **b, c** Regenerated isotropic-flexible brown NCs-GO and black NCs-rGO hybrid films. **d, e** XRD pattern and FTIR spectra of NCs, NCs-GO, and NCs-rGO nanocomposites. Reproduced with permission from Ref. [32] Copyright © 2018, The Royal Society of Chemistry

Remarkably, green and non-toxic GO direct reduction reagents such as L-ascorbic acid (Vitamin C) and hydroiodic acid (HI) have been proved to be more effective on direct GO in-situ chemical reduction. For both reagents, well dispersion and high binding nanocomposite have been demonstrated to lead to flexible and high conductive hybrid films [39, 126, 137]. The chemical reduction mechanism can be interpreted by the conversion of GO nanosheets oxygen-functional groups to CO_2_/CO and H_2_O formation under specific conditions of high temperature and a noble gas. Comprehensive work by Chen et al. [32] has demonstrated the green fabrication potential of smart hybrid nanocellulose-graphene hybrid films. Through facile and green approach by dissolving NCs and dispersing GO homogeneously in conventional alkaline–aqueous urea solution following by in-situ chemical reduction of rGO thru L-ascorbic acid (Vitamin C) solution as eco-friendly reduction reagent as illustrated in Fig. 12a–c [137–140]. The obtained isotropic-flexible NCs-GO/rGO hybrid films demonstrated an improvement in the mechanical and electrical properties proportionally with GO content (2–8 wt%). Interestingly, the smart NCs-GO/rGO hybrid film displays multifunctional high sensitivity abilities for different mechanical, environmental, and bio-human signals considered it as multi-sensing platform. Herein, it should be indicated that the enhancement of NCs-GO/rGO nanocomposites homogeneity attributed to the strong interfacial interaction of hydroxyl groups between the well-dispersed rGO nanosheets and dissolute NCs matrix. The generated XRD diffraction pattern confirms this and FTIR spectra shifted broadband at (3360 cm^−1^) of NCs and (3365 cm^−1^) of GO toward a new band at (3330 cm^−1^) of NCs-GO and (3335 cm^−1^) of NCs-rGO (Fig. 12d, e) [32].

**Fig. 13:**
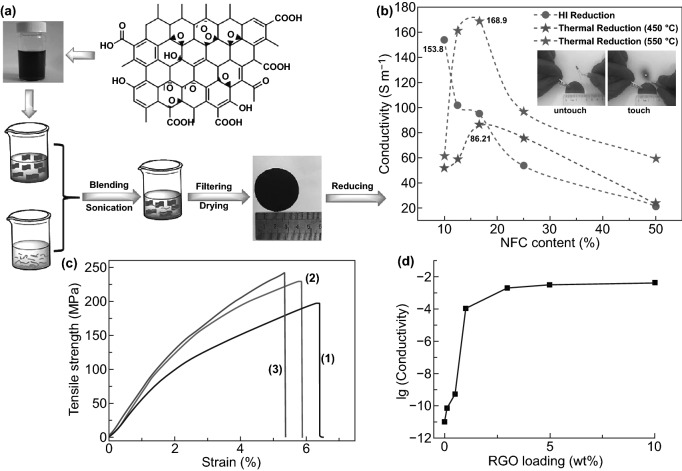
**a** Schematic view of the synthesizing, functionalization, and fabrication process of CNFs-GO/rGO hybrid nanocomposite film. **b** Influence of green GO chemical and thermal reduction and CNFs content on the electrical conductivity of CNFs-rGO hybrid film. Reproduced with permission from Ref. [37] Copyright © 2020, Springer. **c** Stress–strain curves of (1) BNCs, (2) GO, and (3) BNCs-GO hybrid nanocomposites films. **d** Electrical conductivity of BNCs-rGO hybrid film. Reproduced with permission from Ref. [142] Copyright © 2011, Elsevier Ltd

Correspondingly, work by Chen et al. [37] demonstrated the efficiency of GO direct chemical and thermal reduction via both green reductant reagents (Vitamin C and HI) combined with CNFs as a green dispersant and reinforcing agent as presented in Fig. 13a [35]. Extraordinarily, the fabricated CNFs-GO/rGO hybrid film was achieved with high electrical conductivity around (168.9 S m^−1^) as a result of the CNFs influence on avoiding poor dispersion and high GO content (Fig. 13b). The authors attributed the morphological and lamellar structural changing phenomenon of CNFs-rGO hybrid film with HI, VC, and thermal reduction to the surface functional groups (i.e., OH, COOH, and C–OH) transformation, deformation, carbonization, and hybridization. In addition to acting as a green dispersant, the large aspect ratio and abundant functional groups of CNFs diminish the binding difficulties with GO/rGO resulting in an excellent conductive network, which improves the electrical conductivity of CNFs-GO/rGO hybrid film [37]. Further to CNCs and CNFs potentials, bacterial nanocellulose (BNCs) [141] also shows a remarkable impact on enhancing mechanical and electrical properties of NCs-GO hybrid nanocomposites through the simple mixing-drying process. Feng et al. [142] have fabricated a flexible and electrically conductive BNCs-GO hybrid film using a simple vacuum filtration technique. Notably, rGO high content (> 1 wt%) by hydrazine, resulting in increased BNCs-rGO hybrid film uniformity and electrical conductivity. The sufficient interactions, unidirectional uniform dispersion, and continuous conductive networks between GO/rGO nanosheets and BNCs matrix are responsible for high Young modulus (1.7 ± 0.2 GPa), tensile strength (242 ± 7 MPa), and electrical conductivity (1.1 × 10^−4^ S m^−1^) enhancement (Fig. 13c, d). Thus, to achieve the desired mechanical and electrical properties of fabricated NCs-GO/rGO hybrid nanocomposites through a simple solution process, a high-quality NCs green aqueous dispersion is essential to prevent GO/rGO nanosheets aggregation and stabilization in different organic–inorganic solutions. However, GO and NC proportions and GO reduction reagents play a vital role in accomplishing a high electrical conductivity of the fabricated NCs-GO/rGO hybrid films. Typically, this is only achievable through designing stable, well dispersion and homogenous hybrid nanocomposites. Even though remarkable growth has already been achieved in the green fabrication through simple mixing-casting-drying techniques of advanced hybrid NCs-GO/rGO nanocomposite using NCs and ILs as a green dispersant, reductant, filler, and stabilizer. However, structural orientation-direction control is a crucial challenge for realizing their high-performance advanced functional hybrid films’ potentials.

### Multilayer Assembly Techniques

Multilayer assembly technique’s key advantage has the nanoscale ability to assemble and generate hybrid organic–inorganic nanocomposites into a layer-by-layer ultrathin film with control of the size, internal organization, and morphology for various applications [41, 143].

**Fig. 14:**
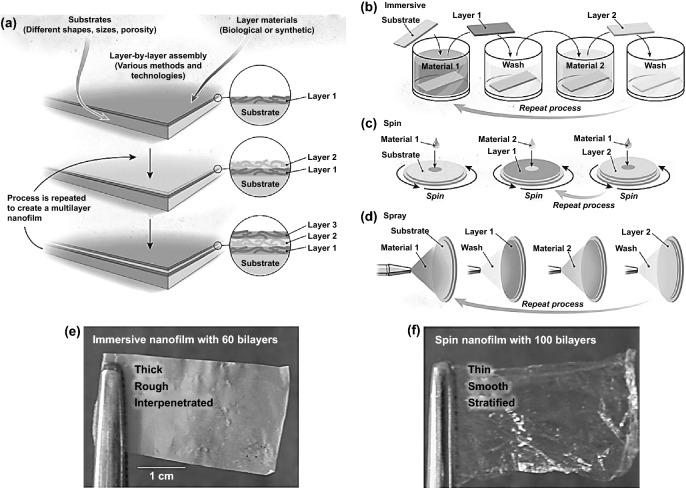
**a** Schematics view of multilayer assembly technique. **b**–**d** Schematics view of the cyclical process of immersive, spin, and spray assembly spray layer by layer assembly. **e, f** Immersive and spin assembled films of hydrophobically modified poly(ethylene oxide) and poly(acrylic acid). Reproduced with permission from Ref. [41] Copyright © 2015, American Association for the Advancement of Science

The prevalent assembling approach for functional hybrid ultrathin films into substrates with different cyclical processes of fabrication hybrid nanocomposites is categorized into an immersive, spin, and spray assembly. These cyclical process of each category contains several steps: beginning with absorbed the first component layer onto the substrate, then washing the substrate before absorbing the second component layer and finally repeating the deposition-coating-spraying and washing process until the desired nanoscale thickness of multilayer film is reached as presented in Fig. 14 [41]. The functionality of GO/rGO nanosheets and NCs matrix is a direct result of the orientation-direction control and alignments through various fabrication techniques [109, 144, 145]. Mainly, multidimensional NCs are relatively easy to orient and uniform by a variety of forces (i.e., van der Waals, electrostatic, and shear forces) due to their anisotropic shape, which may lead to enhance the hybrid nanocomposites mechanics, optics, and swelling behavior. Herein, we focus on the multilayer assembly techniques relevant for the eco-synthesis hybrid nanocomposites with multi-sensing potentials, i.e., that have attracted attention and linked to their multifunctionality. Recently, several NCs-GO hybrid nanocomposites have been fabricated via multilayer assembly technique process driven by numerous electrostatic forces [106] and synergistic interfacial interactions [105]. However, GO nanosheets and NCs chains nano-dispersion and planar orientation are considered key challenges in preparing and fabricating hybrid NCs-GO/rGO ultrathin films. Consequently, there is a necessity for assistant dispersant and stabilizer agents to improve dispersion, orientation, and homogenization of the NCs-GO hybrid nanocomposites.

**Fig. 15:**
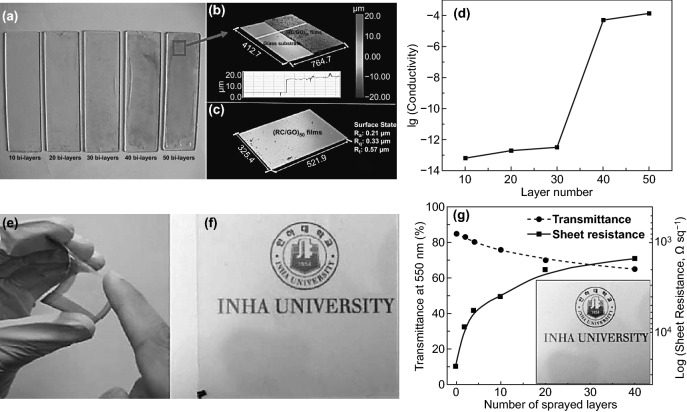
**a** (RC/GO)_*n*_ ultrathin film surface topography of different bilayers (*n* = 10–50). **b, c** Thickness and roughness of (RC/GO)_50_ nanocomposite film on a glass substrate. **d** The layers’ influence on the electrical conductivity of (RC/GO)_*n*_ composite film. Reproduced with permission from Ref. [105] Copyright © 2012, Chinese Materials Research Society, Production and hosting by Elsevier B.V. **e**, **f** Flexibility and transparency of CNCs-rGO hybrid film. **g** The sprayed CNCs-rGO layers resistance via conductivity measurements. Reproduced with permission from Ref. [146] Copyright © 2014 WILEY‐VCH Verlag GmbH & Co. KGaA, Weinheim

As aforementioned in Sect. 4, ILs are considered to be an ideal one for both GO/rGO and NCs. Tang et al. [105] have successfully fabricated thin regenerated hybrid cellulose/GO ultrathin film by multilayer immersive assembly with the assistance of [Bmim]Cl ionic liquid. The overall cyclical immersive-deposition and washing process into glass substrate of dispersed GO and dissolved cellulose in [Bmim]Cl generated multilayer (RC/GO)_*n*_ film to desired (*n*) thickness. Figure 15a–c displays the high level of smoothness and homogeneity of (RC/GO)_50_ thin film (400 nm), which refers to the strong interfacial bonding during deposited-washed cycles. The high GO content and multilayer number, as demonstrated in Fig. 15d, have enhanced the thinner-highly ordered GO/cellulose film’s electrical conductivity (1.3 × 10^–4^ S m^−1^), which makes it a promising candidate for sensors and electrochemical devices. The applicability of multilayer spraying assembly was reported by Sadasivuni et al. [146], which have fabricated transparent and flexible CNCs-rGO hybrid nanocomposite film with chemical synthesis assistance of isophorone diisocyanate (IPDI) reagent. The strong synergetic interfacial interactions were demonstrated due to the (IPDI) organic reagent reaction mechanism as GO functionalizing agent and coupling agent between GO nanosheets and the CNCs matrix. The functionality of (IPDI) reagent (amino, isocyanate, and diisocyanate functional groups) besides the standard anhydrous hydrazine GO reduction process enhances cross-linking and binding CNCs-GO layers, resulting in flexibility, high optical transparency, and proximity sensing capability (Fig. 15e, f). The number of (CNCs-rGO)_*n*_ layers (*n* ≈ 40) through cyclical spraying-coating assembly on polymer substrate has a significant impact on the electrical conductivity with an inverse relationship between the optical transparency and resistivity of fabricated hybrid film (Fig. 15g). Hence, the equilibrium between the conductivity and transparency of optimum multilayer (CNCs-rGO)_*n*_ hybrid film is desirable for optoelectronic sensors’ future applications.

**Fig. 16:**
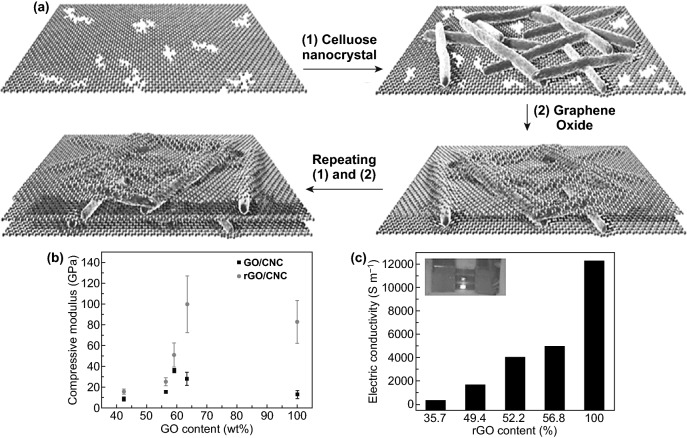
**a** Cyclical process of CNCs and GO multilayer assembly strategy. **b** Micromechanical properties of CNCs-GO and CNCs-rGO films with different GO content. **c** rGO content impact in electrical conductivity. Reproduced with permission from Ref. [106] Copyright © 2015, WILEY‐VCH Verlag GmbH & Co. KGaA, Weinheim

Hybrid organic–inorganic nanocomposite most critical issue is how to reinforce nanoscale constituents [111] to achieve superior mechanical properties, including stiffness and toughness with high performance [17]. However, minor deformation originated from the weak interfacial interactions and dispersion in hybrid nanocomposite leading to inferior mechanical properties [147, 148]. As mentioned previously in Sects. 2 and 3, the GO nanosheets and NCs chains as classical reinforcing nanocomponents have high binding potential and strong interfacial interactions could tackle and eliminate this deformation drawback and gain high performance of GO-NCs hybrid nanocomposites. By utilizing multilayer spin assembly technique to combine flexible two-dimensional GO nanosheets and stiff rod 1D CNCs, Xiong et al. [106] have well-fabricated multilayer CNCs-GO hybrid ultrathin film by cyclical deposition spinning strategy as illustrated in Fig. 16a. The outstanding mechanical performance of ultra-robust highly ordered multilayer hybrid ultrathin film could be attributed not only to the sufficient modified CNCs-GO surface charge and unique synergistic interfacial interactions but also to the effective stress transfer applied by modified spin assembly with high-speed centrifugal force. Moreover, electrochemical GO reduction boosted the micromechanical properties to the high level (i.e., Young modulus boost up to around 169 ± 33 GPa (Fig. 16b), due to strong *π–π* interactions and tight interlayer spacing [106]. As obviously evident as a common benchmark for NCs-GO hybrid nanocomposites, rGO high content displays a significant impact on CNCs-rGO hybrid ultrathin film’s electrical conductivity in Fig. 16c.

**Fig. 17:**
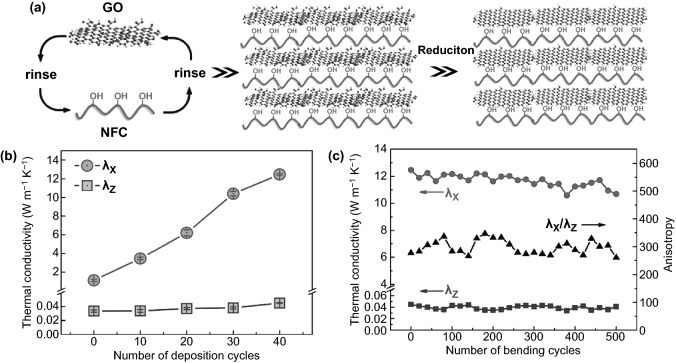
**a** Schematic view of preparation and fabrication of (CNFs-rGO)_*n*_ hybrid film by multilayer deposition assembly on a flexible CNF substrate. **b** Thermal conductivity of (CNFs-rGO)_*n*_ hybrid film with (*n*) cyclical deposition on a flexible CNF substrate. **c** (CNFs-rGO)_*n*_ hybrid film in-planer (*λ*_*X*_) and cross-plane (*λ*_*Z*_) under bending cycles. Reproduced with permission from Ref. [96] Copyright © 2017, American Chemical Society

Alongside free-standing, flexibility, and high electrical conductivity of fabricated NCs-GO/rGO hybrid ultrathin films, thermal conductivity and shielding from excess heat are gain more attention for thermal management of hybrid nanocomposites films. Driven by the multilayer assembly technique potentials of directing-controlling macro–micro–nanostructures [41] as well as strong interfacial interactions between CNFs and GO, Song et al. [96] have fabricated flexible and highly anisotropic thermally conductive CNFs-rGO hybrid film through multilayers deposition assembly as shown in Fig. 17a. The highly ordered fabricated CNFs-rGO hybrid film by assembly technique owing to nano-level orientated and well-aligned layered structures of 2D GO nanosheets and 1D CNFs rigid solid substrate, which leads to superior in-planer (*λ*_*X*_) thermal conductivity [12.6 W m^−1^ K^−1^] and inferior cross-plane (*λ*_*Z*_) thermal conductivity [0.042 W m^−1^ K^−1^] values at 40 deposition cycles (Fig. 17b). However, the strong hybridized anisotropy [94, 149–152], as shown in Fig. 17c, and rGO low content (1.0 wt%) in (CNFs-rGO)_40_ hybrid film display a high efficiency on enhancing thermal conductivity in comparison with other high rGO content hybrid nanocomposites [152–154]. The widely reported electrospinning multilayer assembly technique also proved to be ideal for fabricating and functionalizing NCs-rGO hybrid nanocomposites. Fu et al. [130] have eco-friendly fabricated a conductive aligned NCs-GO hybrid film by the electrospinning-deposition assembly of acetate CNFs-rGO. The super resistivity, flexibility, and durability of strain sensor are attributed to structural alignment and strong interfacial interaction between hydrophobic rGO conductive layers and acetate CNFs hydrophilic substrates.

In this context, multilayer assembly might be a powerful technique to combine most of the desirable mechanical, electrical, optical, and thermal properties in fabricating hybrid ultrathin films for various applications, including multi-sensing. However, the GO/rGO content influence on the fabricated hybrid ultrathin films’ electrical and thermal conductivity should be considered, besides adjusting NCs content to maintain high mechanical properties.

## Multifunctional Sensing Platform

Current research interest into NCs-GO/rGO hybrid nanocomposites, particularly for developing multifunctional hybrid films as the multi-sensing platform, is growing rapidly, attributable to their stabilizing characteristics, functionalization merits, and outstanding desirable properties. Typically, well-designed, eco-synthesized, and green fabricated hybrid nanocomposites films with desirable mechanical, electrical, and thermal properties are the key factors for various applications such as multi-sensing. This section focused on the most recent advanced NCs-GO/rGO hybrid films used in multi-sensing applications and considered as promising multifunctional sensing platform.

An ideal multifunctional hybrid film should be easy to fabricate, cheap to manufacture, and able to detect signals with high selectivity and sensitivity. As pointed out in previous sections, NCs-GO/rGO hybrid nanocomposites can eco-friendly synthesized and regenerated into hybrid films with outstanding mechanical properties, high electrical and thermal conductivity. Consequently, the combination of these aspects makes NCs-GO/rGO hybrid film attractive for multi-sensing applications. In most cases, NCs-GO/rGO hybrid films are applied as a multifunctional sensor that can be responding to multiple stimuli such as mechanical (stress–strain, pressure, and deformation) or environmental (temperature, humidity, and liquids-solvents) and other human bio-signals (human motion and breathing). Also, combined NCs and GO/rGO are used as one of the components that accompany by other stimuli-responsive compounds used in the fabrication of sensors. The multifunctional NCs-GO/rGO hybrid films exhibit a wide multi-sensing range in responding to diverse stimuli through different sensing mechanisms (i.e., relative capacitance or resistance measurements), which will be discussed in the following subsections.

### Mechanical and Environmental Signals

**Fig. 18:**
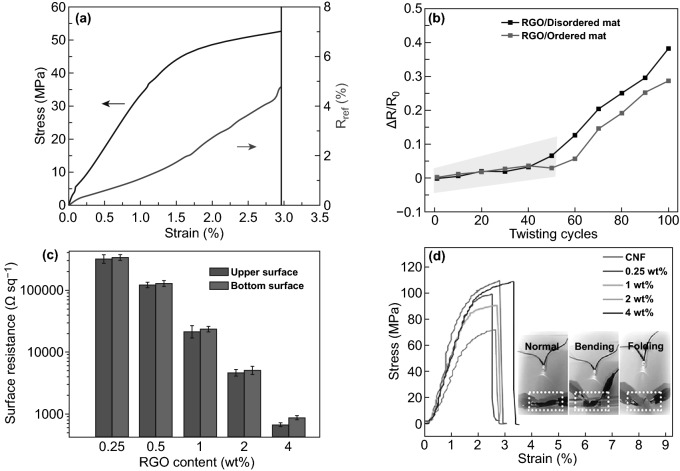
**a** NCs-rGO hybrid film electrical resistivity under external stress–strain. Reproduced with permission from Ref. [32] Copyright © 2018, The Royal Society of Chemistry. **b** Resistance change of CNFs-rGO hybrid film under twisting cycle numbers. Reproduced with permission from Ref. [130] Copyright © 2018, IOP Publishing Ltd. **c**, **d** Sheet resistance and stress–strain curves of CNFs-rGO hybrid film with different rGO content (0.25–4 wt%). Reproduced with permission from Ref. [155]. Copyright © 2018, American Chemical Society

The mechanical signals detection of free-standing, flexibility, and high electrical conductivity multifunctional NCs-GO/rGO hybrid films is based on the strong reported synergistic interfacial interactions between NCs matrix network and GO/rGO nanosheets. Accordingly, the fabricated NCs-GO/rGO hybrid film electrical resistivity can change upon mechanical impacts such as stress–strain changes. Chen et al. [32] investigated strain sensing of hybrid NCs-rGO film by the relative electrical resistance (*R*_rel_) changing under strain with cyclic loading 1 mm min^−1^ up to fracture. Remarkably, to some extent, the hybrid NCs-rGO film shows a linear strain response (4.7% *R*_rel_ at 2.9% strain) until the fracture, as illustrated in Fig. 18a. In another study, Fu et al. [130] developed a strain sensor based on conductive rGO and acetate CNFs hybrid nanocomposite film. The results showed that applied twisting cycles (100) to the CNFs-rGO hybrid film could change the electrical resistance (Δ*R*/*R*_0_) (Fig. 18b). Noticeably, Hou et al. [155] have reported that rGO content and CNFs anisotropy proved to be a significant factor in the electrical sensitivity toward stress–strain changes, as shown in Fig. 18c, d.

**Fig. 19:**
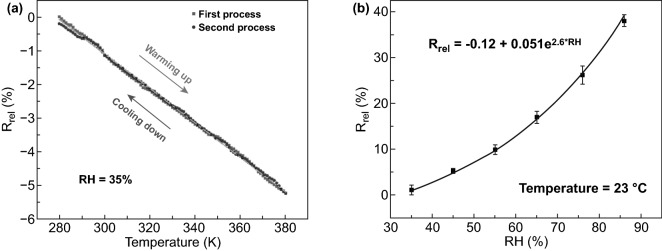
**a** Hybrid NCs-rGO film temperature sensitivity by (R_rel_) changes in the temperature range of 280–380 K. **b** NCs-rGO hybrid film humidity sensitivity in a variation of (RH: 30–90%). Reproduced with permission from Ref. [32] Copyright © 2018, The Royal Society of Chemistry

Recently, NCs-GO/rGO hybrid film sensitivity toward changing environmental conditions like temperature and relative humidity (RH) correlated with relative resistance–capacitance has confirmed based on the GO/rGO nanosheets high electrical conductivity [12, 31, 37] and anisotropic NCs matrix swelling–shrinkage behaviors and absorption–desorption capabilities [1, 4, 20, 57, 109]. In this regard, Chen et al. [32] have proved the high temperature and RH sensitivity of NCs-rGO hybrid film by the relative electrical resistance (R_rel_) changing when applied for RH variations of (30–90%) and temperature sweeps [280 →  ← 380 K] of warming up and cooling down cycles (Fig. 19a, b). The evolution of the (*R*_rel_) mechanism at constant *T* 23 °C, RH 35%values can be referring to NCs matrix swelling behavior because of water molecules absorption at the rGO surface, which leads to change the distance and destruct the conductivity pathways between rGO nanosheets.

**Fig. 20:**
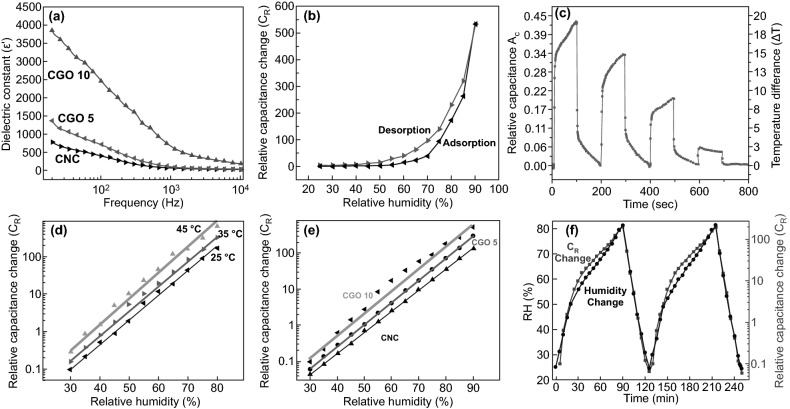
**a** Dielectric behavior of NCs-GO/rGO hybrid film with frequency variation. **b** Absorption–desorption behavior and humidity hysteresis of NCs-GO/rGO hybrid film. **c** NCs-GO/rGO hybrid film temperature sensitivity limit by relative capacitance changes. **d**–**f** Relative capacitance change (*C*_R_) of NCs-GO/rGO hybrid film with different temperatures, RH, and time. Reproduced with permission from Refs. [31, 34]. Copyright © 2015, 2016 Elsevier B.V

Comprehensive work in the Jaehwan Kim group has demonstrated the high temperature and relative humidity sensitivity of NCs-GO/rGO hybrid films based on their dielectric behavior (Fig. 20a) and absorption–desorption capabilities (Fig. 20b) [31, 34]. Based on the efficient interactions of rGO nanosheets conjugated and non-conjugated networks within the NCs matrix, the sensing and detecting mechanism of cyclical heating and cooling temperature in range (25–80 °C) and RH in range of (25–90%) is correlated with dielectric response and relative capacitance change (*A*_C_–*C*_R_) (Fig. 20c–f). The reasonable numbers of oxygen-containing functional groups on the NCs-GO electrode-dielectric interfaces result from thermal annealing reduction of GO [156, 157], optimized the tunneling conduction and charge carrier transport via interfacial dielectric polarization effects. Consequently, this electrical transmission optimization is responsible for the hybrid film fast response by relative capacitance during the charging-discharging of cyclical heating and cooling (interfaces bonding spacing, charge density, and dielectric relaxation time) [158]. Furthermore, NCs’ hydrophilicity and absorption–desorption behavior and the inverse relation between temperature range and RH interpreted the different responses in relative capacitance with time for a different size and NCs/GO content (wt%). Using CNCs-GO/rGO hybrid nanocomposites, the Jaehwan Kim group further demonstrated the high relative humidity sensitivity [34]. The extensive interfacial interactions between CNCs and GO interfaces induced the dielectric behavior and reinforced the Maxwell–Wagner polarization in the hybrid nanocomposite [156, 159]. The relative capacitance change (*C*_R_) results in Fig. 20d–f efficiently show the linear and rapid relative humidity sensitivity of CNCs-GO/rGO hybrid film with a similar tendency over time. This tendency is linked to the low humidity hysteresis represented in the good absorption–desorption behavior under applied reversible RH in the range of (25–90%). The proposed humidity sensing mechanism of CNCs-GO/rGO hybrid film: CNCs surface hydroxyl groups able to detect humidity as well as GO plane–center–edges hydroxyl and carboxyl groups able to attract more water molecules. However, different environmental conditions could impact the T-RH sensor’s performance and should take into consideration to minimize the measurement error. NCs have shown a significant role in enhancing the NCs-GO/rGO hybrid film’s sensitivity and selectivity by their swelling–shrinkage behavior to different solvents. On the basis of the NCs stability and anisotropic orientation in fabricated NCs-GO/rGO hybrid film besides the NCs swelling–shrinkage behavior, which widens its multi-sensing range to liquids-solvents with different types, temperature, and concentrations.

**Fig. 21:**
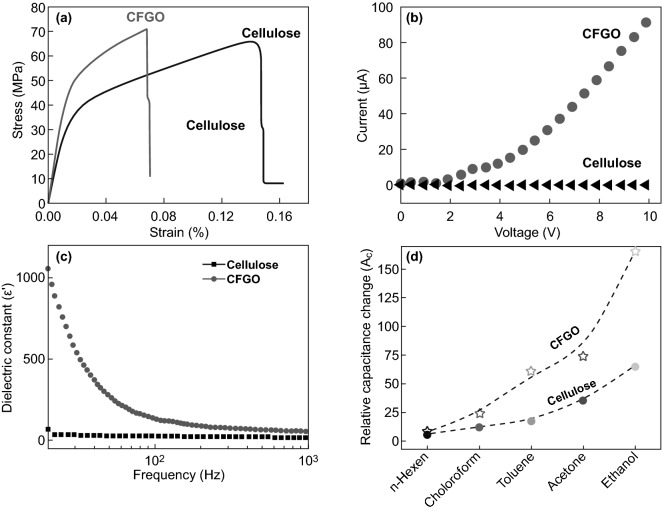
**a**–**c** Mechanical, electrical, and dielectric properties of NCs-GO hybrid film and **d** Relative capacitance change (*A*_C_) of NCs-GO hybrid film with several solvents. Reproduced with permission from Ref. [33] Copyright © 2015, Elsevier B.V

**Fig. 22:**
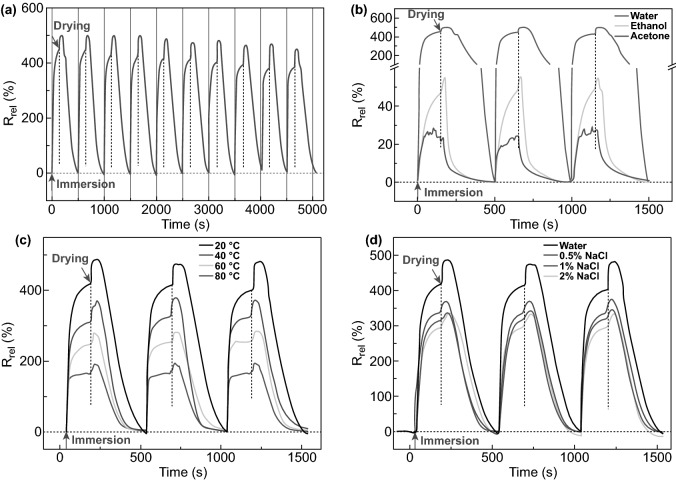
**a**–**d** Hybrid NCs-rGO film relative electrical resistance (*R*_rel_) changes with time during immersion and drying cycles in water, different solvents, water at different temperatures, and aqueous NaCl solutions (20 °C) at different salt concentrations. Reproduced with permission from Ref. [32] Copyright © 2018, The Royal Society of Chemistry

To expand the application of NCs-GO hybrid films as a multi-sensing platform, the Jaehwan Kim group has validated the different solvents sensing and detection ability depends on the desirable mechanical, electrical, and dielectric properties, as illustrated in Fig. 21a–c [33]. The fabricated NCs-GO solvent sensor exhibits extremely sensitive responses to organic solvents such as Toluene, Acetone, and Ethanol (Fig. 21d) depended on diffusion mechanism by capacitance change due to the GO nanosheets spacing distance caused by NCs swelling chains. The free charge motion at interfacial polarization besides the Maxwell–Wagner polarization reinforcement is suggested to be responsible for the sensing mechanism. Built on this concept, Chen et al. [32] group went one step further to demonstrate the efficiency of NCs-rGO hybrid film for liquids-solvents detection of ions and concentration based on the relative electrical resistance (R_rel_) changes. Interestingly, the NCs-rGO hybrid film was found to be a multifunctional liquids-solvents sensor during immersion and drying cycles with different solvents, temperatures, and salt concentrations (Fig. 22a, b). Evidently, R_rel_ changes significantly when immersion and drying cycles were applied in water under several temperatures (20–80 °C) and different salt concentration solutions (0.5–2%) (Fig. 22c, d). The sensing mechanism behind the remarkable sensitivity and selectivity in water and other solvents as ethanol and acetone refer to the differences in NCs swelling–shrinkage behavior to each liquid on rGO nanosheets surface, which consistentity change the electrical resistivity with conductivity disconnection between rGO nanosheets. Moreover, the liquids sensitivity is strongly being influenced by liquids temperature and concentrations.

### Human Bio-signals

**Fig. 23:**
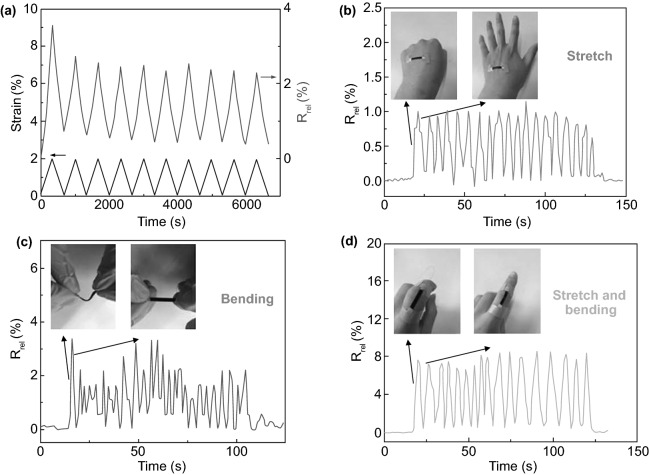
**a** NCs-rGO hybrid film electrical resistivity (*R*_rel_) under fixed cycling stretching strain. **b**–**d** NCs-rGO hybrid film electrical resistivity (*R*_rel_) under stretching-releasing, bending-relaxing, and combined stretching-bending of human hand motion. Reproduced with permission from Ref. [32] Copyright © 2018, The Royal Society of Chemistry

**Fig. 24:**
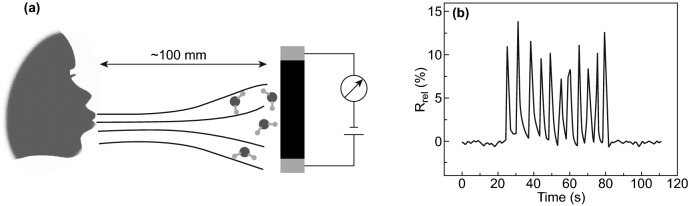
**a** Schematic view of in-situ monitoring of human breathing cycle by NCs-rGO hybrid film (100 mm distance from the nose). **b** Evolution of (*R*_rel_) during inhalation and exhalation cycles. Reproduced with permission from Ref. [32] Copyright © 2018, The Royal Society of Chemistry

The outstanding mechanical properties of NCs-GO/rGO hybrid film are represented in lightweight, bendability, and flexibility, besides the GO/rGO high electrical-thermal conductivity and NCs matrix absorption–desorption abilities of molecules, broaden its multi-sensing range to bio-sensing and detecting bio-signals like human hand motion and human breathing cycles. Chen et al. [32] have found that NCs-rGO hybrid film able to detect repeated strain cycles (Fig. 23a) generated from human hand motions such as stretching-releasing and bending-relaxing. The relative electrical resistance (*R*_rel_) fluctuation (Fig. 23b-d) of the flexible hybrid film fixed on the human hand corresponded well to the different strain rates generated from various motions and positions. Importantly, the NCs-rGO hybrid film demonstrated an exceptionally dynamic response to various temperature-humidity conditions by (*R*_rel_) evolution, which allows it to detect human breathing during inhalation and exhalation cycles (Fig. 24a, b). The effective and quick response mechanism of NCs-rGO hybrid film to different human hand motions and human breathing cycles is attributed to the repeatable change of distance and conductivity between rGO nanosheets and NCs matrix revocable moisture absorption and hygroscopic swelling behavior.

Currently, driven innovation in intelligent human–machine interactions and digital human healthcare [160] is attracted attention by green fabricating integrated short-range proximity sensors to detect bio-signals without physical contact with different proximity sensing mechanisms [160–163].

**Fig. 25:**
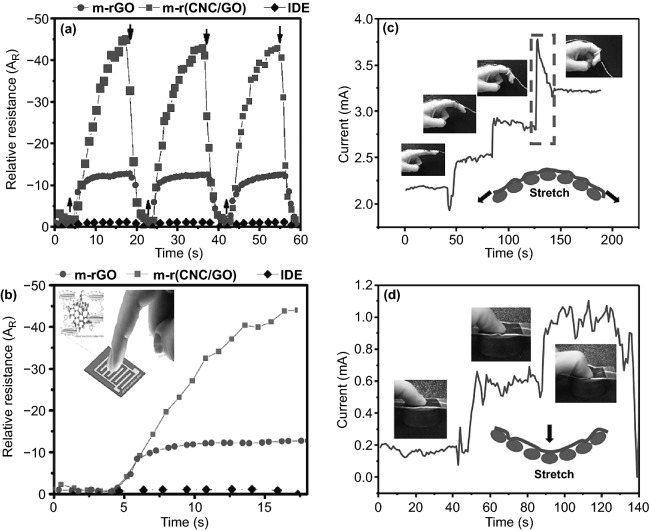
**a** Proximity sensitivity of CNCs-rGO hybrid film at 0.2 mm human finger distance. Reproduced with permission from Ref. [146] Copyright © 2014, WILEY‐VCH Verlag GmbH & Co. KGaA, Weinheim. **b** Tactile sensitivity of CNCs-rGO hybrid film. Reproduced with permission from Ref. [164] Copyright © 2015, Society of Photo-Optical Instrumentation Engineers (SPIE). **c**, **d** Resistance change of CNFs-rGO strain sensor in monitoring human finger bending and pushing motions, respectively. Reproduced with permission from Ref. [130] Copyright © 2018, IOP Publishing Ltd.

The NCs-GO/rGO hybrid film display interesting, innovative features for proximity sensing depended on their balance between high optical transparency and electrical-thermal conductivity. By varying the surface-volume ratio along with charge-storage capacitance with respect to synergetic interfacial interactions, NCs-GO/rGO hybrid film with highly proximity sensing abilities could be accomplished. Successfully, Sadasivuni et al. [146] have developed transparent and flexible CNCs-rGO hybrid film as a proximity sensor for human finger detecting and human skin recognition by resistance change under controlled conditions [146, 164]. The nonlinear and semiconductor behavior of CNCs-rGO hybrid film, as a direct influence of CNCs interference into rGO nanosheets conducting network, resulting in the highly resistive hybrid film. In this work, the short-distance proximity sensing mechanism through relative resistance can be attributed to the disruption of the highly CNCs-rGO uniformly charged concentrated electrostatic field [163] by external stimuli (human finger approaches 2 mm distance, 30% RH and 25 °C) as shown in Fig. 25a, b. Herein, the high sensitivity and fast response likely refer to the 3D porous network structural merits with effective charge transport of CNCs-rGO macroscopic hybrid film. Therefore, NCs-GO/rGO hybrid film exhibits synergistic sensing characteristics to the human finger as a proximity sensor. A further demonstration of in-situ monitoring and detecting human finger motions was achieved by Fu et al. [130] through developing a strain sensor based on acetate CNFs substrate and conductive rGO hybrid film. The authors found that once the CNFs-rGO hybrid film fixed along with the human finger, it became more sensitive with linear response to bending and pushing motions (Fig. 25c). Also, CNFs-rGO hybrid film showed a better response to the finger pressure (Fig. 25d).

Interestingly, the fast strain sensitivity response, stretchability, flexibility, and durability of NCs-GO/rGO hybrid film as multifunctional strain sensor recommend it for monitoring and detecting different bio-signals from the human body motion like muscles. As evidently mentioned, the NCs-GO/rGO hybrid film sensitivity and selectivity of diverse liquids under different conditions as temperature and ion concentration expand its applications for wearable multifunctional smart sensors to monitoring human body healthcare by detecting sweat and tracking the ion concentration. In this regard, Xu et al. [165] group went a step further to validate NCs-rGO hybrid film’s proficiency for in-situ monitoring of human motion and sweat by electrical resistance and colorimetric changes.

**Fig. 26:**
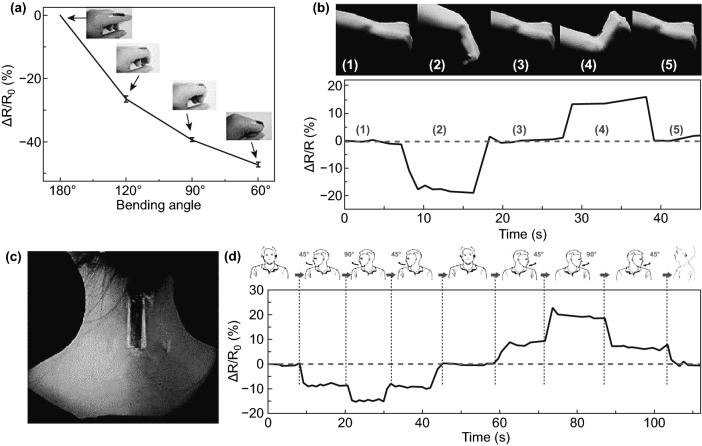
**a**–**d** Electrical resistance changes of multifunctional NCs-GO hybrid film sensor attached to different parts of the human body to detect and monitor various human motions of the human forefinger, wrists up-downward, and head rotation (0°–45°–90°) under multidirectional motions, separately. Reproduced with permission from Ref. [165] Copyright © 2018, The Royal Society of Chemistry

In this work, a highly sensitive NCs-rGO hybrid multifunctional wearable sensor film was successfully fabricated based on the hybridization of rGO conductive film and acetyl NCs flexible substrate. The HI reduction of hydrophilic GO nanosheets resulted in free-standing hydrophobic rGO nanosheets owing to the elimination of GO surface–center–edges oxygenated functional groups. The synergetic effects between rGO and acetyl NCs lowered the deformability (i.e., high elastic modulus: 2.4–4.1 × 10^3^ MPa) and rGO nanosheets conjugated structures have elucidated the flexibility and conductivity of the NCs-rGO hybrid film. Moreover, rGO hydrophobicity prevents the fabricated hybrid film sensor from bio-signals interference, achieving simultaneous in-situ monitoring. More profoundly, the electrical resistivity (–/ + Δ*R*/*R*_0_) consistently changed with microstrain deformation variations and multidirectional human motions (under compressive and tensile state) with small hysteresis and reliability (Fig. 26).

**Fig. 27:**
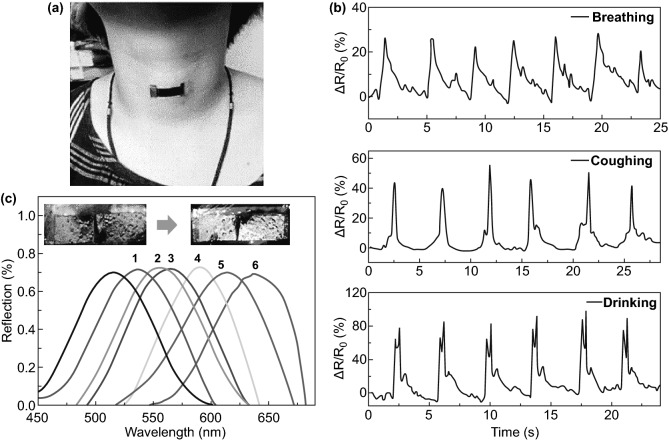
**a** In-situ monitoring and detection of human throat motions and sweat during motion. **b** Electrical resistance (Δ*R*/*R*_0_) change patterns of human throat laryngeal prominence under different motions as breathing, coughing, and drinking. **c** Reflection spectra of sensor detection human artificial sweat (1–6: 30–680 mM NaCl concentrations). Reproduced with permission from Ref. [165] Copyright © 2018, The Royal Society of Chemistry

Consequently, this is proved the sensor high strain response and wide detection range of various human body motions, including finger bending-stretching, wrists upward-downward, small-scale throat motions, and head rotations (Fig. 27). The authors claimed that the reflection spectra result, as shown in Fig. 27c, confirmed the obtained NCs-rGO hybrid film sensor high resistivity and reliability to human sweat during motions and different NaCl consternation (30–680 mM) with structural colorimetric change (blue-green/yellow-green) or diffraction-peak shifts. Thanks to the anisotropic NCs absorption behavior and mesoporous ordered structure of such hybrid sensor, more sweat can absorb and store into mesopores, which could be beneficial for human health evaluation through the body sweat and dehydration.

## Summary and Outlook

Herein, a brief overview of the most advanced novel NCs-GO/rGO hybrid functional films, including synthetization, functionalization, fabrication, and implementation as a multifunctional sensing platform, was highlighted. First of all, we introduced some fundamental conceptions of nanocellulose and graphene top-down synthetization routes with unique properties and outstanding features. Then, by utilizing these unique properties in green solvent aqueous suspensions (i.e., nanocellulose, ionic liquids), improved functionality of NCs-GO/rGO hybrid nanocomposites are possible. After that, through eco-friendly and facile approaches, different techniques have been applied to fabricate NCs-GO/rGO hybrid films and enhanced their properties. Finally, to well understand of these NCs-GO/rGO hybrid films’ characteristics and outstanding desirable properties, their multi-sensing applications were discussed.

Significantly, in this review, we have demonstrated the nanocellulose abilities to act as a multifunctional green dispersant, stabilizer, filler, and reductant agent for graphene derivatives, resulting in superior rheological, mechanical, electrical, thermal, and optical properties. Also, the nanocellulose surface was proven to act as a unique functional platform with controlling the self-assembly of graphene sheets, which in turn considered a promising accomplishment for multi-sensing applications. However, some critical challenges should be addressed and taken into consideration to fully realize nanocellulose-graphene hybrid films as realistic multi-sensing platforms in practical aspects. The first and foremost challenges refer to the nanocellulose and graphene origin sources top-down synthesis standardization and characterization of nanoscale dimension structures, surface functional groups, and assembling behaviors. Nevertheless, the large-scale empirical development of NCs-GO/rGO hybrid films can provide a deeper understanding and detail technical datasheets for commercial aspects in the near future. Consequently, practical applications of such advanced hybrid functional materials can open prospective and attract more attention as next-generation advanced functional materials with high performance, eco-effective, and cost-effective. Also, graphene derivatives are commonly considered non-cytotoxic and biocompatible. However, their synthetization routes extremely influence the in vivo and in vitro assessments as results of the residual reagents/solvents (i.e. hydrazine) utilized during synthetization process, which can interact with tissues/cells and induced cytotoxicity. Hence, NCs with multifunctional eco-synthesis routes abilities, besides the eco-friendliness merits such as non-toxicity, biocompatibility, and biodegradability can significantly eliminate and diminish these induced cytotoxicity and adverse side effects.

Future outlooks consist of the collaboration of engineers and biomaterial scientists to elaborate marketable and accessible products of such promising hybrids for multi-sensing applications. For instance, a multifunctional wearable NCs-GO/rGO hybrid film sensor exhibited excellent high strain response and wide detection range of various human body motions, including finger, wrists, throat motions, and head rotations, besides the human sweat ion concentrations during the motion. These multifunctional-integrated smart sensors can closely measure, mimic, and in-situ monitor a broad range of bio-physical, bio-chemical, and environmental signals that offer critical insights into overall human health status and driven innovations in digital healthcare like multifunctional wearable healthcare monitors and so forth.
